# Hyper-CEST NMR of metal organic polyhedral cages reveals hidden diastereomers with diverse guest exchange kinetics

**DOI:** 10.1038/s41467-022-29249-w

**Published:** 2022-03-31

**Authors:** Jabadurai Jayapaul, Sanna Komulainen, Vladimir V. Zhivonitko, Jiří Mareš, Chandan Giri, Kari Rissanen, Perttu Lantto, Ville-Veikko Telkki, Leif Schröder

**Affiliations:** 1 Molecular Imaging, Department of Structural Biology, Leibniz-Forschungsinstitut für Molekulare Pharmakologie (FMP), 13125 Berlin, Germany; 2 Division of Translational Molecular Imaging, Deutsches Krebsforschungszentrum (DKFZ), 69120 Heidelberg, Germany; 3 NMR Research Unit, University of Oulu, 90014 Oulu, Finland; 4 Research Unit of Medical Imaging, Physics and Technology (MIPT), University of Oulu, 90014 Oulu, Finland; 5 University of Jyvaskyla, Department of Chemistry, 40014 Jyväskylä, Finland

**Keywords:** Organometallic chemistry, Self-assembly, Molecular self-assembly, Chemical physics, Solution-state NMR

## Abstract

Guest capture and release are important properties of self-assembling nanostructures. Over time, a significant fraction of guests might engage in short-lived states with different symmetry and stereoselectivity and transit frequently between multiple environments, thereby escaping common spectroscopy techniques. Here, we investigate the cavity of an iron-based metal organic polyhedron (Fe-MOP) using spin-hyperpolarized ^129^Xe Chemical Exchange Saturation Transfer (hyper-CEST) NMR. We report strong signals unknown from previous studies that persist under different perturbations. On-the-fly delivery of hyperpolarized gas yields CEST signatures that reflect different Xe exchange kinetics from multiple environments. Dilute pools with ~ 10^4^-fold lower spin numbers than reported for directly detected hyperpolarized nuclei are readily detected due to efficient guest turnover. The system is further probed by instantaneous and medium timescale perturbations. Computational modeling indicates that these signals originate likely from Xe bound to three Fe-MOP diastereomers (*T*, *C*_3_, *S*_4_). The symmetry thus induces steric effects with aperture size changes that tunes selective spin manipulation as it is employed in CEST MRI agents and, potentially, impacts other processes occurring on the millisecond time scale.

## Abstract (web summary)

Self-assembling nanostructures that trap guest molecules find applications in materials research and the life sciences. Here, authors describe how ultra-sensitive NMR reveals sub-types with broken symmetry that significantly impacts guest exchange.

## Introduction

Self-assembled metal organic cages are supramolecular nanostructures that are widely used as hosts for various guests with applications as synthetic ion channels^1^, sensors for organic analytes^2,3^, in molecular recognition^4,5^, drug delivery^6,7^, catalysis^8^, gas adsorption and separation^9^, and magnetic resonance imaging (MRI)^10,11^. Besides the three spatial dimensions, time is an important fourth dimension for the overall parameterization of cavity shape and guest exchange kinetics in cages and metal organic frameworks (MOFs)^12,13^. Tailored host cavities have a crucial impact on the capacity and selectivity for guests^14,15^. The time dimension is directly linked to guest turnover and the control of, e.g., magnetic properties of guests or catalysis mediated by supramolecular chemistry^16^. The involved stereochemistry is an important parameter as it impacts stereoselective transformations, chiral recognition of reversibly bound guests and steric fine tuning of the host portals. Metal organic polyhedra (MOPs) are one class of such nanostructures and they generally allow formation of hosts with broken symmetry, albeit some conformers can be highly favored over others. A mixture of different isomers is usually obtained upon constructing MOPs from asymmetric ligands. The application potential can be greatly influenced by the type of isomer and thus knowledge about the distribution and the availability of certain isomers is an important aspect. As an example, certain isomers from a mixture can exhibit a significantly lower IC_50_ value for treatment of different cancer cells than others^17^. Additionally, resolved isomers of enantiopure MOPs are applied in asymmetric catalysis and chiral recognition, respectively^18,19^. Also, a chiral amorphous MOP applied as the stationary phase in gas chromatography (GC) was recently shown to separate different enantiomers and various types of organic compounds^20^. In general, NMR spectroscopy (^1^H and ^15^N NMR^21^) is suitable to reveal symmetry differences but requires relatively high concentrations and is unsuitable for dilute, short-lived species. NMR signals can be enhanced by hyperpolarization (e.g., via dynamic nuclear polarization, DNP) in solution state NMR^22^. However, such DNP at room temperature still requires mandatory spin-labeling of the analyte during synthesis and 10^17^ spins in 10^−7^ L sample volume. Even this enhancement is insufficient for highly dilute states, particularly when somewhat rapid exchange is involved.

**Fig. 1:**
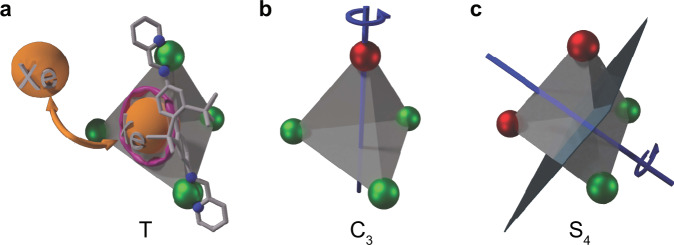
Schematic illustration of Fe-MOP and its three diastereomers (atoms not to scale). Only one of the ligands oriented along the tetrahedral edge complexing two Fe^2+^ cations is shown for clarity purpose. Depending on how the participating nitrogen atoms (shown in blue) arrange around the metal centers (shiny spheres), the portal size and shape is slightly deformed (illustrated by the pink blurry ring). This impacts Xe exchange kinetics. Schematics of different Fe-MOP diastereomers and their symmetry axes/planes for the *T* (**a**), *C*_3_ (**b**), and *S*_4_ (**c**) diastereomers. **a** The *T* isomer has the ligands coordinated to homochiral metal centers (only green colored Fe^2+^ metal nodes) via the *anti*-linkages. **b**, **c** Conversely the ligands coordinate to the heterochiral metal centers (mix of red and green colored Fe^2+^ metal nodes) in *C*_3_ and *S*_4_ schemes via the *syn*-linkages.

Due to such limitations, certain conformations might have been overlooked in MOPs which can exist as tetrahedral cages^23–36^ constructed from octahedral complex-forming metal ions. These hosts can temporarily bind different hydrophobic guests^37–40^. Some MOP ligands generate considerable fractions of heterochiral (*C*_3_) or achiral (*S*_4_) cages^41^ that are guest-responsive and exhibit dynamic interconversion between the diastereomers^21^. However, many initially synthesized M_4_L_6_ cages (M and L refer to metal and ligand) have been reported to exhibit only homochiral (*T*) species in solution. An example is the water-soluble tetrahedral [Fe_4_L_6_]^4−^ cage formed by treating 4,4’-diaminobiphenyl-2,2’-disulphonic acid and 2-formylpyridine in water together with Fe(II) and a base^42^. Experimental evidence (e.g., ^1^H and ^15^N NMR) is available exclusively for its *T* species (Fig. 1a). Recent studies on a series of related Co-containing MOPs^43,44^ also found no signs for any other versions.

Conformations with broken symmetry but sparse population could be undetectable albeit such conformers in combination with a certain exchange regime would indeed cause a considerable amount of guests to experience a certain type of host cavity over time. Such inclusion complexes might, however, survive long enough to have an impact on how a guest probes different symmetries and thus also allow for stereoselective recognition of reactants that are processed in tailored cages. Apart from guest recognition, the overall host symmetry is linked to the spatial arrangement of the ligands and enables fine tuning of the portals. This can have great impact on exchange kinetics. A related aspect is the field of MOFs where the presence of metastable intermediate states in such dynamic structures is in certain cases only observable after loading such structures with gaseous guests or by controlling temperature and pressure^12,45^. The flexibility limits of most reported self-assembling systems is underexplored^13^ and understanding the consequences of fundamental properties like broken symmetry that may impact guest exchange mechanisms in flexible frameworks is key to the successful development of applications. Sensitive tools to monitor all cavity conformations and their guest uptake and release are therefore of high importance for understanding and designing these intriguing systems.

We thus explored ultra-sensitive NMR saturation transfer with ^129^Xe to search for additional conformations of a yet exclusively assumed homochiral host. Xe as a monoatomic, spherical guest serves as a neutral “spy” to explore multiple symmetry environments. Hyperpolarized (HP) ^129^Xe can be easily dispersed into solution and saturated magnetization is readily replaced by on-the-fly delivery of fresh HP gas. The experiment can be repeated within a few seconds to allow for stepwise acquisition of well-resolved chemical shift information and/or signal averaging. Even though this type of spectroscopy unlike multi-dimensional NMR does not directly reveal structural information on the cavity seen by the guest atom, differences in the Xe exchange timescales become visible in the line shape of the saturation responses. Exchange kinetics are highly relevant in host-guest systems with non-covalent nature where Xe can serve as a model guest to explore cavity access. The noble gas is a powerful hyperpolarized NMR reporter because it can be more or less directly applied to bulk material and sense exchange kinetics in host sites without the need for cumbersome isotope labeling of the cavity-shaping atoms but rather provides a reversibly bound spin label itself to probe the existence of multiple conformations.

We investigated the water-soluble tetrahedral [Fe_4_L_6_]^4−^ cage by performing chemical exchange saturation transfer (CEST) NMR experiments using HP ^129^Xe^46,47^ since Xe forms inclusion complexes with this Fe-MOP^48^. Combining the sensitivity enhancement of HP nuclei^49^ with CEST, i.e., hyper-CEST, yields a ca. 10^5^ - 10^6^ fold signal enhancement over conventional NMR^50–54^. Recently, the ^129^Xe hyper-CEST technique was applied for demonstrating a significant enhancement of the NMR signal of dissolved gas using a water soluble MOF^55^. In another recent study, the capability of ^129^Xe hyper-CEST NMR was demonstrated for differentiating a complex mixture containing MOFs as nano-cages in which the latter displayed similar chemical compositions. This was possible by tuning the MOF structure and by encoding multiple false colors for observed Xe chemical shifts^56^. Saturation transfer (ST) techniques are a powerful tool that find an increasing number of applications. The magnetization in the bound pool is saturated by utilizing a continuous wave rf pulse applied over a time that is significantly longer than the average residence time of transiently bound spins. During this saturation period, chemical exchange causes a signal loss in bulk pool by affecting hundreds to thousands of spins per exchange site, thereby leading to accumulation of destroyed magnetization in the detection (bulk) pool^51^. ST techniques improve NMR sensitivity, retain the spectral dimension^57^, and can be “tuned” in sensitivity to different time scales of exchange processes. Furthermore, Xe significantly extends the detection range towards exchange rates of 10^4^ Hz due to its large chemical shift range^58^. This enables detecting so far elusive host conformations of stereochemistry interest.

In this work, the Xe@Fe-MOP system is investigated by ^129^Xe NMR under different perturbation conditions (e.g., temperature cycling, portal blocking, and guest competition). We observe multiple signals of HP ^129^Xe interacting with Fe-MOP and validate normal cage integrity for guest access. The exchange pathways that Xe undergoes between different chemical environments of Fe-MOP and solution are checked further by inversion recovery (IR) and 2D CEST (short saturation) measurements. The multiple responses in the CEST spectra are interpreted using complementary theoretical methods and first principles modeling as computational methods are considered an integral part to understand such systems^13^.

## Results and discussion

### Thermal stability of supramolecular Fe-MOP

**Fig. 2:**
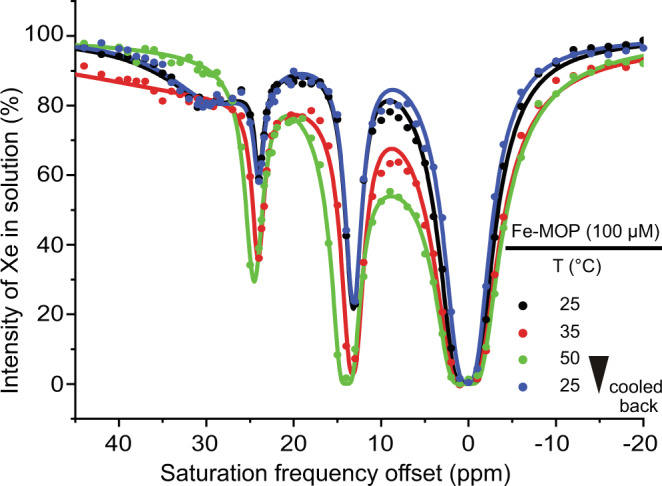
Thermal stability of Fe-MOP studied at variable temperature using ^129^Xe-hyper-CEST. Fe-MOP (100 *μ*M) at 25 ^∘^C showed 3 responses at 13, 24, and 30 ppm. Stepwise increase in temperature causes two signals (13, 24 ppm) to increase while the third peak (30 ppm) disappeared at *T* ≥ 35 ^∘^C. Cooling the Fe-MOP solution back to 25 ^∘^C restored the initial result.

Tetrahedral Fe-MOPs ([Fe_4_L_6_]^4−^[(CH_3_)_4_N]_4_^+^) were self-assembled under basic conditions at 50 ^∘^C (Supplementary Fig. 1 and Section 3)^42^. The product was structurally characterized using ^1^H-NMR and ESI-MS (Supplementary Figs. 2 and 3). Fe-MOP (100 *μ*M) encapsulation of Xe was investigated by Xe hyper-CEST NMR at temperatures ranging between *T* = 25 ⋯ 50 ^∘^C (see Supplementary Section 3.2, for additional tests between 4 and 20 ^∘^C). Beside the direct saturation signal of free Xe in water at 0 ppm, the CEST spectra displayed three responses at 13, 24, and 30 ppm, respectively, at 25 ^∘^C (Fig. 2; it is common practice in CEST experiments that the chemical shifts are referenced with respect to the signal that is observed, i.e., in this case, the signal of free Xe in solution). This is in contrast to only one peak corresponding to the 13 ppm signal of bound Xe observed using direct ^129^Xe NMR detection^48^. Also, the Co-containing versions [Co_*n*_Fe_(4−*n*)_L_6_]^(4−)^ (*n* = 1–3) have been reported to display only one signal each for encapsulated Xe^44^. With increasing temperature, the intensities of the narrow signals at 13 and 24 ppm increased while the broad one at 30 ppm disappeared into the baseline upon reaching 35 ^∘^C. When reducing *T* from 50 ^∘^C back to 25 ^∘^C, the data matched the initial result (Fig. 2 and Supplementary Fig. 4 together with Supplementary Video 1). We thus conclude that the 30 ppm signal represents already relatively fast exchange at room temperature (RT), which yields inefficient CEST conditions at higher temperatures. The two stronger signals, however, represent intermediate exchange at RT, which yields even better saturation transfer at higher exchange rates. Overall, the temperature cycling revealed the structural robustness of the self-assembled Fe-MOP via reproducible hyper-CEST signatures.

### Examining the origin of multiple CEST peaks

Next, the reason for observing multiple CEST peaks was checked. No indication was found that the oxidation state of the Fe(II) centers changed while the iron was participating in coordination with the ligands (see Supplementary Fig. 6). Likewise, the presence of residual amounts of acetone used during Fe-MOP recrystallization cannot be the origin of additional CEST signals; in fact, large excess of (CH_3_)_2_CO causes reduced intensity, see Supplementary Fig. 6. We also checked if storage of Fe-MOP in solution might have led to deterioration. However, CEST spectra of freshly mixed Fe^2+^ cations and ligands revealed instantaneously the previously observed signals. The response at 0 ppm appears broadened and a somewhat matching observation applies to the signal at 30 ppm (Supplementary Fig. 7). We interpret this as a presumably less efficient initial locking of the gas reflected by accelerated exchange until a chemical equilibrium is reached.

Another option for explaining multiple CEST signals is the contribution of different optically resolved (D (ΔΔΔΔ) and L (ΛΛΛΛ)) Fe-MOP isomers. The protocol for resolving the racemic (*rac*) mixture^18^ is described in Section 6 of the Supplementary information. The CEST spectra indicate that both D- and L-isomers contribute with the same type of hyper-CEST responses (Supplementary Fig. 9).

**Fig. 3:**
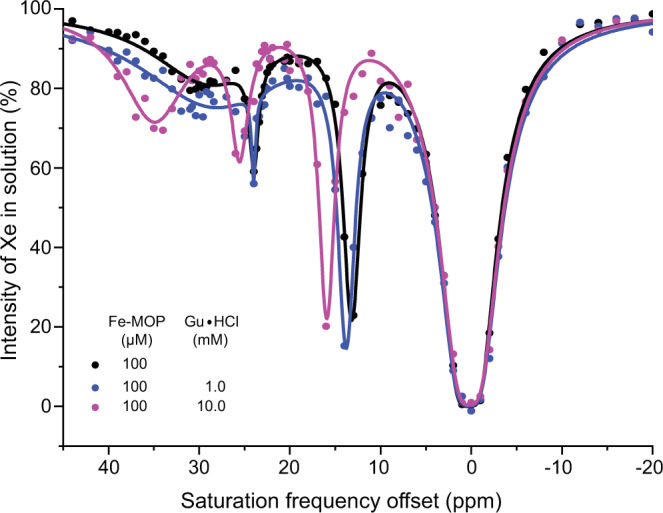
Access of Xe to Fe-MOP cages under variable blocking of the faces by Gu ⋅ HCl. The CEST peaks of Fe-MOP (100 *μ*M) shifted slightly upon treatment with 1 mM Gu ⋅ HCl. 10 mM Gu ⋅ HCl resulted in more spectral shift due to higher salt concentration and slowed down the Xe exchange kinetics for the 30 ppm signal.

Next, we altered the access of Xe to the cages by a blocking strategy acting outside the cavity. The access through the four faces of Fe-MOP was impeded using excess guanidinium hydrochloride (Gu ⋅ HCl) which is known to alter the incoming guest kinetics for cyclohexane^59^. Here, Fe-MOP (100 *μ*M) was gradually blocked by increasing the Gu ⋅ HCl concentration. The CEST spectra remained unaffected for [Gu ⋅ HCl] < 1 mM. At 1 mM Gu ⋅ HCl, a slight left shift of CEST peaks was observed (somewhat anticipated due to higher salt concentration). A 10 mM concentration of Gu ⋅ HCl finally resulted in more left shifted peaks while presumably slowing down the Xe exchange for the signal at 30 ppm, which becomes better resolved (Fig. 3). Xe is supposedly small enough to retain sufficient cavity access. Even high concentrations of Gu ⋅ HCl can only decelerate the exchange, primarily affecting signals that reflect already enhanced exchange under normal conditions.

### Challenging the Fe-MOP cavity with larger competing guests

**Fig. 4:**
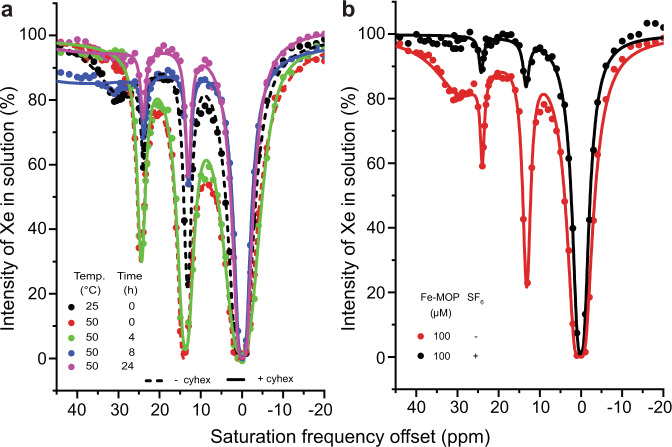
Challenging the capture of Xe in Fe-MOP by competing larger guests. **a** The reference spectrum of Fe-MOP (100 *μ*M) plus cyclohexane at 25 ^∘^C revealed 3 CEST peaks. At 50 ^∘^C, the CEST peak at 30 ppm vanished into the baseline and intensities of 2 CEST peaks (13, 24 ppm) were approaching the baseline over 24 h indicating more cyclohexane encapsulation than Xe. **b** The larger competing guest, SF_6_, caused even stronger decrease in the observed CEST responses.

Assuming sufficient purity and integrity of the hosts, we challenged the interaction of Xe with Fe-MOP directly inside the cavity through competition by adding guests like cyclohexane and SF_6_. Fe-MOP (100 *μ*M) was mixed with cyclohexane (150-fold excess) and a reference CEST spectrum for no cyclohexane encapsulation was acquired at 25 ^∘^C (Fig. 4a). The Fe-MOP cavity was then probed by Xe in the presence of cyclohexane at 50 ^∘^C over 24 h. The slowly progressing cyclohexane encapsulation was imminent after 8 h as it led to reduced CEST peak intensities. Subsequently, Xe was removed by degassing and the sample mixture containing cyclohexane was maintained at 50 ^∘^C for 24 h. Then, re-measuring the sample at 50 ^∘^C with Xe displayed ca. 56% and 45% reduction of the 13 and 24 ppm CEST peak intensities compared to Fe-MOP alone (Fig. 4a; the 30 ppm signal is anyway hardly identifiable at 50 ^∘^C). Similarly, the Fe-MOP cavity was challenged by encapsulating an even larger and stronger binding guest, SF_6_^60^. Measuring the SF_6_ ⊂ Fe-MOP (100 *μ*M) at 25 ^∘^C after 3 days of introducing the SF_6_ atmosphere at 50 ^∘^C showed ca. 62 % and 31 % reduction in the intensities of the 13 and 24 ppm CEST peaks compared to untreated Fe-MOP. The fast exchanging CEST peak at 30 ppm disappeared into the baseline after the SF_6_ treatment even when measuring the sample at 25 ^∘^C (Fig. 4b). Altogether, Xe binding remains possible owing to its smaller size and incomplete competing guest encapsulation (e.g., ~ 75% for SF_6_^60^). Here, the CEST technique reveals otherwise missed residual encapsulation. These results illustrate that the newly discovered signals must originate from cavity environments and that not only Xe has access to these.

### Exchange pathways between different CEST pools

We next revisited direct HP Xe NMR spectroscopy at fairly high Fe-MOP concentration (10.3 mM) and could show a previously unknown second and third Xe@Fe-MOP peak at 24 and 30 ppm, respectively (Supplementary Fig. 11a, b). This enabled us to estimate the populations of the suspected additional species (Supplementary Fig. 11c). The relative populations of the 13, 0 ppm (solution pool), 24 and 30 ppm pools at 10.3 mM cage concentration estimated from the direct spectrum are 1, 0.32, 0.010, and 0.0017, respectively (see Supplementary Fig. 11c). Despite their low population, these are acting as rather efficient CEST pools and are indicative for relative fast exchange rates. The strongest peak represents ca. 5.6 mM of occupied MOPs (see Supplementary Information), which is ca. 55 % of the overall host concentration.

**Fig. 5:**
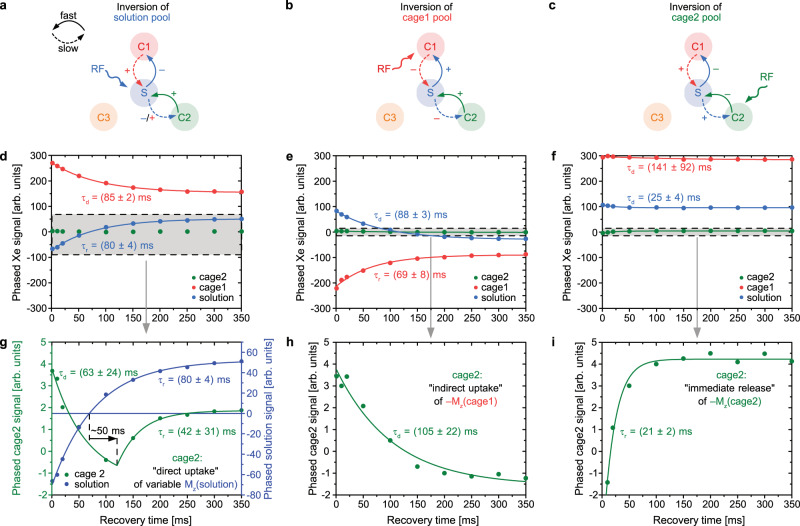
Selective inversion recovery studies of Fe-MOP (10.3 mM) at 25 ^∘^C. **a**–**c** Pool network and detectable exchange routes. In each pairwise on/off exchange there is a fast rate constant from the smaller into the larger pool and a slow release constant vice versa. The directly inverted pool is indicated by the RF label. +/− signs indicate the net impact of enabling a signal recovery (growth) or a signal decay (as reaction to uptake of inverted magnetization). **d**–**f** Time evolution of the magnetizations. All can be fit to mono-exponential laws. The time constants *τ*_*d*_ (decay) and *τ*_*r*_ (recovery) give a qualitative impression for ordering direct (faster) and indirect (slower) responses. Equilibration between C1 and S happens on a medium time scale. The directly inverted pool always reacts fastest. **f** C1 reacts rather slow to C2 inversion while S sees the rather fast release from C2. **g**–**i** Zoom into the time curves for C2 illustrating three different regimes. **g** The perturbation from S propagates with medium speed into C2 because it simultaneously equilibrates with C1. C2 changes its behavior ca. 50 ms after the directly connected magnetization in S changes sign (piecewise fit for C2 with variable transition point). This reflects the direct uptake of the variable S magnetization. **h** Perturbation of C1 propagates only indirectly into C2, as reflected by a slow uptake of negative magnetization. **i** Directly inverted C2 signal is immediately released with rather fast exchange into S. Its uptake in S is partially compensated by positive magnetization from C1.

In addition, we carried out inversion recovery (IR) experiments at conditions where the solution peak and the two most prominent cage signals can be observed, while one of these three signals was selectively inverted (Fig. 5 and Supplementary Fig. 12 together with Supplementary Video 2). The two bound Xe signals at 13 and 24 ppm were arbitrarily assigned as Cage1 and Cage2, respectively. Regarding this initial subset, the data supports a three site exchange model (Cage1 <-> Solution <-> Cage2), in which the two more intense cage signals were directly connected with the solution pool, but not directly connected with each other (Section 10.1, Supplementary information).

The IR measurements at high concentrations sense the immediate flux of magnetization after a selective, instantaneous perturbation of one pool. They showed that the system equilibrates within ca. 350 ms (Supplementary Movie 2). CEST measurements with detecting pools other than the solution pool provides complementary insights at increased sensitivity for intermediate timescale fluxes between the pools. This must be detected before saturated Xe accumulates in the solution pool and then spreads throughout the network. We thus investigated CEST spectra at 25 and 10 ^∘^C with global readout (Supplementary Fig. 13). A short saturation (*t*_sat_ = 250 ms) was used for measuring the exchange with limited sensitivity enhancement. Saturating the solution peak at 25 ^∘^C yields a strong signal for Cage2 and a weak response for Cage1. This is because the small Cage2 pool is immediately saturated from the bulk pool whereas Cage1 represents a large enough pool that is not completely exchanged within 250 ms. The solution peak showed a (rather weak) response to Cage2 saturation and a decent response for Cage1 saturation. However, Cage2 saturation yields an over-proportional strong CEST response for the solution pool. This again reflects the rather efficient exchange from Cage2 into the solution pool. As Cage1 does practically not respond to Cage2 saturation, we conclude that the flux via the solution pool has not yet reached Cage1 within 250 ms. For the reverse direction, data suggests that despite the indirect flux of saturation from Cage1 via the solution pool into Cage2, this is indeed detectable after 250 ms and comparable to direct flux from the solution pool to Cage2 (Supplementary Fig. 14).

We then cooled the Fe-MOP solution to 10 ^∘^C for decelerated exchange from Cage3 signal and employed a higher saturation strength (*B*_1_ = 5 *μ*T) for a length (*t*_sat_) of 250 ms. Only the solution peak reacted to Cage3 saturation. This suggests that either it is very inefficient pathway or too fast flux is observed from Cage3 into Cage1 or Cage2 (Supplementary Fig. 14b, 15). Overall, the 2D CEST experiment enabled us to establish the possible CEST network between highly and sparsely populated CEST pools and the respective Fe-MOP states.

### CEST spectra simulation and quantification

After excluding Fe-MOP structural degradation and hyper-CEST artifacts (CEST responses were observed without significant changes even after several months and were eventually also observed with thermally polarized Xe; see Supplementary Fig. 16) or structural imbalance induced by Xe bubbling (Supplementary Fig. 10) as explanation, we further analyzed the multiple CEST peaks. We hypothesize that they originate from Xe interaction with three different Fe-MOP diastereomers (*T*, *C*_3_, *S*_4_). Literature strongly suggests that only *T* is observable in solution using conventional detection methods^42,61^. Hyper-CEST, however, made it possible to detect such “hidden states” with low populations, although the ^1^H signals of the minor diastereomers were covered by the stronger signals of the dominating *T* diastereomer (Supplementary Section 14). It is important to note that CEST extends the range that NMR can cover on the time scale of guest exchange kinetics and thus contributes to the need for methods that include time as an important fourth dimension for implementing novel applications with MOFs/MOPs^13^. Even though we did not vary the saturation conditions for the spectra, adjusting the saturation time and power makes CEST with xenon a versatile tool: it covers processes that are too fast for conventional NMR detection as well as those kinetics that accumulate only over 10s of seconds in the bulk pool as the HP ^129^Xe NMR signal has a long life time to store information that is amplified through saturation transfer from a dilute pool. The observation that the CEST signals appear more or less instantaneously (Supplementary Fig. 7) indicates that the sub-populations of locked MOPs form in parallel and that the synthesis does not allow straightforward separation (or interconversion) of them for further isolated investigation. Experimentally obtained z-spectra can be described in the frame of the proposed four site exchange model (Supplementary Fig. 18) in combination with a simulation of the CEST experiment at 25 ^∘^C. Simulations were done by numerical integration of Bloch-McConnell type equations with chemical exchange terms and provided a z-spectrum which fitted excellent to the experimental data (Supplementary Fig. 17).

The simulation gave the following populations of sites for a Fe-MOP concentration of 100 *μ*M: *P*(F) = 96 % > *P*(*T*) = 3.8 % > *P*(*C*_3_) = 0.14 % > *P*(*S*_4_) = 0.041 %. The 96% fraction of free Xe (F) corresponds to 369 *μ*M dissolved Xe at 25 ^∘^C, thus, the readily detectable CEST signal at 30 ppm represents only 160 nM of Xe@*S*_4_. In fact, the pool size of ^129^Xe experiencing the *S*_4_ cavity is then limited to 42 nM. This translates to only 3.8 × 10^13^ detectable spins for the *S*_4_ pool in an 1.5 mL sample, thus demonstrating the enormous sensitivity of the applied technique (10^4^-fold less spins than in DNP NMR^22^). The relative populations of the encapsulated sites are slightly different from those detected from direct spectra under different conditions where the Fe-MOP concentrations exceeds the free Xe concentration. The quantitative analysis showed significant differences in the Xe release rates, ranging from 4 × 10^3^ s^−1^ for the sparsely populated *S*_4_ conformer to 2.7 s^−1^ for the well known *T*-cage. These numbers agree with the qualitative changes in the spectra observed upon heating the sample. This “exchange tuning” aspect that the symmetry brings into the parameter space of MOPs extends the options for designing future hyper-CEST MRI reporters that can also be selectively addressed through sufficient spectral dispersion.

### Computational approach and findings

Quantum chemical calculations of ^129^Xe NMR chemical shifts were performed (see *Computational methods* section) for Xe atoms placed inside of the three individual diastereomers. The otherwise unknown molecular structures of *C*_3_ and *S*_4_ diastereomers were derived from the X-ray structure^42^ of the *T* diastereomer. The chirality of one or two metal centers was manually changed to create *C*_3_ or *S*_4_ diastereomer, respectively. The conformational space of each isomer was sampled by generating high number of conformers (see Methods section below and Supplementary Section 13). The lowest free energy (*G*) values for conformations of the *C*_3_, and *S*_4_ are Δ*G* = 0.6 and 43.0 kJ/mol, respectively, with respect to the lowest energy conformation of *T* diastereomer. The thorough scrutiny of the full conformational space indicate that the lowest *G* conformations are the plausible structure candidates. This is supported further by large free energy differences to the next lowest conformations of each diastereomer (Δ*G* = + 4 ⋯ + 9 kJ/mol) as well as high energy barriers between them.

Despite the density functional theory (DFT) computed ^129^Xe chemical shifts for a few low energy structures of the conformational search are overestimated (see Section 13.3 in Supplementary material), there is an obvious trend of *δ*(*T*) < *δ*(*C*_3_) < *δ*(*S*_4_). The energetically most plausible lowest *G* structures also follow this trend and, therefore, their structures were studied further. DFT geometry optimization increases their volumes (118.4, 105.7, and 96.0 Å^3^ for *T*, *C*_3_, and *S*_4_, respectively; Xe van der Waals volume^62^: 44 Å^3^). The cages seem to relax around the Xe atom during the optimization in liquid environment, thereby leading to a decrease in cavity volume compared to X-ray data.^42^ Larger cavities by DFT optimization results in ^129^Xe chemical shifts within the experimental chemical shift range (see Table 5 in Supplementary material). The best estimates of ${\delta }^{{}^{129}{{{{{{{\rm{Xe}}}}}}}}}$ were obtained by statistical thermal averaging over Xe motion at room temperature. For the lowest *G* conformation of *T* diastereomer, the obtained *δ*(*T*) = 14.1 ppm (solvent shift w.r.t gas shift is 188 ppm) matches well with the experimental value of 201 ppm. Values for other diastereomers, *δ*(*C*_3_) = 66.7 ppm and *δ*(*S*_4_) = 114.5 ppm, follow the above-mentioned chemical shift trend. Obviously, chemical shift differences are much larger than those obtained from the CEST spectra. However, currently neglected finite temperature effects due to motion of the cage atoms as well as solvent molecules may well decrease the chemical shift differences between the three diastereomers.

Therefore, the computational modeling suggests that the lowest CEST signal (13 ppm with respect to Xe in water) arises from the well-studied *T* diastereomer, while the 24 and 30 ppm signals may originate from *C*_3_ and *S*_4_ diastereomers, respectively.

For *S*_4_ diastereomer, there are nine conformations with large variation in the volume while under similar energy criteria, our sampling approach exhibited only two and three conformations for *T* and *C*_3_ cavities, respectively. This indicates that the structure of *S*_4_ diastereomer is more flexible thus allowing larger openings for accommodation of different guests with varied sizes by its cavity. This finding potentially indicates easier and faster in-out exchange of Xe and may explain the specific features of the 30 ppm CEST signal: Firstly, the broader signal (see Fig. 1) is a demonstration of the faster exchange process. Secondly, the signal disappeared above 35 ^∘^C, as a consequence of the transition from intermediate to fast exchange regime. Thirdly, the chemical shift of the signal responds more to the temperature cycling than other signals (see Fig. 1 and Supplementary Fig. 4), and the CEST signal becomes significantly narrow at low temperatures, denoting the transition between exchange regimes.

Theoretically, the additional CEST signals might arise also from cages loaded with several Xe atoms (see *Computational methods* section). However, loading the much larger solid-state X-ray cavity of the *T* diastereomer^42^ with two Xe atoms increased the calculated chemical shift of about 400 ppm compared to loading single Xe atom (accommodating three and four Xe atoms resulted in even larger chemical shifts). Therefore, we can exclude this explanation. Similarly, we were able to exclude the possibility that the additional signals could arise from simultaneous encapsulation of Xe with N_2_ or He, which were present in the hyperpolarized gas mixture; computational estimates resulted in much larger chemical shift changes than the experimentally observed values and signatures of additional signals were observed even in the thermally polarized experiments without N_2_ and He. Finally, we expect that, due to its hydrophobicity, the cage does not encapsulate water molecules together with Xe; loading of the *T* diastereomer with Xe and H_2_O resulted also computed chemical shifts, which are much higher than the experimentally observed shifts.

In conclusion, we obtained experimental evidence through Xe NMR for two previously unknown bound Xe signals (24 and 30 ppm) for a self-assembling Fe-MOP. Xe even retained accessibility to the cavity upon impeding the portals by excess Gu ⋅ HCl, albeit with slower exchange. Competition with larger and stronger binders such as cyclohexane and SF_6_ considerably reduces ( ~ 30–60 %) the transient binding of Xe but also demonstrates that other guests have access to the additionally identified cavity environments. The Xe exchange pathways between different CEST pools were established by IR and 2D CEST experiments and the exchange was found to occur presumably all via the solution pool. This indicates that Xe interaction with different structural forms (diastereomers) of Fe-MOP might be the reason for observing multiple, consistent and reproducible CEST peaks. The hypothesis was supported through extensive first principles modeling showing that structural differences of diastereomers explain both the order of observed Xe chemical shifts and the variety in exchange kinetics. This study demonstrates that the molecular symmetry is linked to different guest exchange regimes with release of bound Xe ranging between 10^3^ s^−1^ and 10^0^ s^−1^. The insights thus address two of the central questions in this field^12,13^: (a) what are the guest exchange time scales occurring in supramolecular nanostructures and (b) what tools are available to observe them? These results illustrate the value of hyperpolarized guests that can be delivered repeatedly within a few seconds to reveal hidden host conformations in detailed solution spectra of bulk material without sophisticated sample preparation. MOP systems thus provide intriguing encapsulation, spin capture, and exchange properties that can be generated by performing selective chemistry either at the metal center (e.g., by metal substitution/exchange) or via the ligand structure (e.g., by post-synthetic ligand exchange, tethering different linker types), respectively. Future studies shall investigate the CEST imaging capabilities of different MOP symmetries and if the time scale that was sufficient for selective RF manipulation inside the formerly hidden conformations would also allow chemical reactions to happen with stereoselective features.

## Methods

### Materials used

4,4’-diaminobiphenyl-2,2’-disulfonic acid hydrate (70 wt.% in H_2_O) was purchased from Santa Cruz Biotechnology, Inc. Heidelberg, Germany. Iron(II) sulfate heptahydrate (≥ 99%), 2-formylpyridine (99%), tetramethylammonium hydroxide pentahydrate (≥ 97%), cyclohexane (≥ 99.8%), ((*S*)-BINOL) i.e., *S*-(-)-1,1’-Bi(2-naphthol), 99 % and Pluronic^®^L-81 were procured from Sigma Aldrich Chemie GmbH, Steinheim, Germany. Guanidinium hydrochloride (Gu ⋅ HCl, ≥ 99,5%), was obtained from Carl Roth GmbH & Co.KG, Karlsruhe, Germany. Sulfur hexafluoride (SF_6_) gas was kindly provided by Prof. Thomas Braun, Humboldt-Universität zu Berlin, Germany. MilliQ water (18.2 MΩ.cm) was used for all sample preparations. All chemicals and solvents were used as received without any further purification.

### General methods

^1^H NMR data of Fe-MOP were recorded in D_2_O. A potential acetone impurity encapsulated by Fe-MOP was removed by repeating the lyophilization cycles and complete removal of acetone was checked by ^1^H NMR. The electron spray ionization mass spectrometry (ESI-MS) of Fe-MOP was recorded in 1:1 H_2_O:MeOH mixture using Orbitrap Fusion (Thermo Scientific) with Xcalibure set up. The ESI-MS was performed by a direct injection (4 *μ*L/min) of the sample and measuring in the negative mode using a Standard-Source Spray (3 kV) without using any gas. The circular dichroism (CD) measurements of Fe-MOP and its enantiomers in H_2_O were carried out using Jasco J-715 spectrophotometer (Jasco, Maryland, U.S.A).

### Synthesis of supramolecular Fe_4_L_6_ (Fe-MOP)

The supramolecular iron-based metal organic polyhedra (Fe_4_L_6_:Fe-MOP) was synthesized (Supplementary Fig. 1) as reported in an earlier publication^42^. Briefly, 4,4’-diaminobiphenyl-2,2’-disulfonic acid (2.03 mmol), 2-formylpyridine (4.06 mmol), tetramethylammonium hydroxide pentahydrate (4.06 mmol) and iron (II) sulfate heptahydrate (1.35 mmol) were dissolved in 25 ml degassed water. After mixing, the reaction mixture appeared as a dark purple solution. Reaction was carried out for 20 h at 50 ^∘^C under argon atmosphere. The final product was isolated as dark purple crystals by slow vapor diffusion of acetone into the aqueous solution. The structure was validated by ^1^H NMR (Supplementary Fig. 2) and ESI-MS (Supplementary Fig. 3) in agreement to earlier reports^42^.

### Xenon hyperpolarization and delivery

The NMR experiments were performed with a 9.4 T Bruker AV400 NMR spectrometer (Bruker Biospin, Ettlingen, Germany) equipped with gradient coils for MR imaging. The excitation and detection of the NMR signal was achieved using a 10 mm inner diameter double-resonance (^129^Xe and ^1^H) probe. A custom-designed continuous-flow polarizer was utilized to produce hyperpolarized (HP) Xe by spin-exchange optical pumping^63,64^. A gas mixture of 2% Xe (26.4 % ^129^Xe natural abundance), 10% N_2_ and 88% He was used unless otherwise noted. The applied total pressure was 4.5 bar abs., thus the dissolved Xe concentration is ca. 369 *μ*M under these conditions. The achieved ^129^Xe spin polarization was approximately 25%. The gas mix was directly bubbled into 1.5 mL of the sample placed inside a 10 mm thick-walled NMR tube by using a spectrometer-triggered bubble dispenser (3.5 bar overpressure) via five fused-silica capillaries (Polymicro Technologies, Molex Incorporated, Caudebec les Elbeuf, France). The bubbling was controlled by using spectrometer-triggered gas-flow regulators (mass flow controllers, Omega Newport, Deckenpfronn, Germany).

### Hyper-CEST spectroscopy

For each data point in the Chemical Exchange Saturation Transfer (CEST) spectrum, the gas mixture was directly bubbled for 12 s into the sample solution (flow rate 0.1 standard liter per minute (SLM)) followed by a 3 s delay to allow the bubbles to collapse. Hyper-CEST spectra were acquired using a continuous wave (CW)-saturation pulse (length *t*_sat_ = 15 s, amplitude *B*_1_ = 5.14 *μ*T). The (CW)-saturation pulses were applied with 1 ppm increments within 45 to −26 ppm spectral frequency offsets. The spectral acquisition was implemented by referencing the observed chemical shifts to the free Xe in solution peak at 0 ppm. Z-spectra were fitted to the exponential of a sum of Lorentzians using OriginPro 2018 (OriginLab, Northampton, MA). Unless otherwise indicated, the spectra of all samples were acquired at 25 ^∘^C.

### Computational methods

Conformational searches for each diastereomers (*T*, *C*_3_, *S*_4_) were carried out for all three stereoisomers (see Supplementary material). First, high number of structures inaccessible from a local minimum by low energy motion were generated by rotating the benzylsulphonate units (5 angles for every unit resulting in 0.24 × 10^9^ rotamers). Then structures with van der Waals (vdW) clashes and other unrealistic conformers were filtered out and obtained plausible structures were minimally solvated with 36 water molecules - one for each oxygen atom in twelve SO${}_{3}^{-}$ groups. The overall charge on each conformer was compensated with four NH${}_{4}^{+}$ counterions on each face of the [Fe_4_L_6_]^4−^ complex and the Xe atom was placed inside its cavity. The structures were then optimized using the semiempirical extended tight binding GFN2-xTB method^65^ via the XTB code (see Code availability statement). In addition to the explicit counterions and water solvent molecules, an implicit GBSA water solvent model was used in the geometry optimization. The aforementioned optimized structures were selected for further calculations only if Xe remained inside the cavity and SO${}_{3}^{-}$ groups were well exposed to the solvent. To obtain free energy estimates of maximum of twenty lowest energy conformers of each diastereomer, they were subjected to vibrational analysis calculation with the XTB code.

Density functional theory (DFT) calculations for the lowest free energy (*G*) conformers of each diastereomer (see more details in Supplementary material and Supplementary Table 5) were carried out with the Turbomole code^66^. Geometries of the three complexes including four NH${}_{4}^{+}$ counterions were computed at the composite B97-3c method^67^ with implicit COSMO solvation model of water^68^.

At the obtained equilibrium geometries, ^129^Xe NMR nuclear shielding calculations were carried out at scalar-relativistic local exact two-component (X2C) level^69^. We used BHandHLYP^70,71^ DFT functional with D4 dispersion correction^72^ and x2c-TZVPall-s(Xe)/x2c-SVPall(other) basis sets together with finite nucleus model^73^. BHandHLYP has been shown to provide best estimates for Xe chemical shifts in several studies of molecular cavities^48,74–77^ and molecules^78–80^. It was observed in those studies that, while the inclusion of SR effects advances the non-relativistic (NR) calculations for Xe shifts, quite small and invariant spin-orbit (SO) relativistic contribution can be neglected. Therefore, SR method like X2C provides high quality results with moderate computational requirements.

While full molecular dynamics treatment of current extensive systems with counterions and large enough explicit solvation is currently not feasible, we focus on treatment of the most important effect, i.e., Xe thermal motion inside the rigid cage. We use the same approach as in previous studies^48,75,76^, in which canonical Monte Carlo (MC-NVT) simulations with *in house* code were carried out on numerical potential energy surface (PES). PES and Xe NMR shift hyper-surface used for averaging were computed at the same level as the equilibrium geometry calculation above (see simulation details and thermal averages in Supplementary Table 5).

The loading effect on Xe chemical shift was studied by placing one to four Xe atoms inside the Xe-cage potential energy surface of *T*-Fe_4_L_6_^48^. The thermal averaging over the dynamics of Xe atoms at *T* = 300 K was obtained using the modified version of the *in house* MC-NVT code, where both *T*-Fe_4_L_6_ cage contribution to potential energy and NMR chemical shift of Xe were piecewise-linearly interpolated from the non-uniformly sampled 3D surfaces^48^. For Xe-Xe interactions the relativistic potential energy^62^ and the non-relativistic effective binary chemical shift functions by Hanni et al.^81^ were used. Therefore, the Xe chemical shift for each Xe atom is a sum of the thermal averages of the Xe-Xe shift from the binary chemical shift and Xe-*T*-Fe_4_L_6_ shift (with respect to free Xe atom).

## Supplementary information

Supplementary Information

Description of Additional Supplementary Files

Supplementary Movie 1

Supplementary Movie 2

## Data Availability

All data generated and analysed during this study are included in this article and its Supplementary Information, and are also available from the authors upon request. The details about synthesis, structural characterization, hyper-CEST spectra under different perturbation conditions, inversion recovery measurements, 2D CEST (short saturation) experiment and computational methods are available as supplementary information.

## Code availability

The code used for computational methods, including in-house MC-NVT python code, is also available from the authors upon request. The semiempirical extended tight binding program package xtb can be assessed using the following link https://github.com/grimme-lab/xtb. The xTB standalone code, Version 6.2. from the above link was utilized for the semiempirical calculations.
