# Supplementary material for: Hyper-CEST NMR of metal organic polyhedral cages reveals hidden diastereomers with diverse guest exchange kinetics
Source: Nat Commun. 2022 Mar 31;13:1708. doi: 10.1038/s41467-022-29249-w (PMC8971460; doi:10.1038/s41467-022-29249-w)
Supplement: Supplementary file 1 — Supplementary Information [file 41467_2022_29249_MOESM1_ESM.pdf]

Supplementary Information
Hyper-CEST NMR of metal organic polyhedral cages reveals hidden diastereomers with diverse guest exchange kinetics

Jabadurai Jayapaul[1,2], Sanna Komulainen[3], Vladimir Zhivonitko[3], Jiří Mareš[3,4], Chandan Giri[5], Kari Rissanen[5], Perttu Lantto[3,*], Ville-Veikko Telkki[3], and Leif Schröder[1,2,*]

[1]Molecular Imaging, Department of Structural Biology, Leibniz-Forschungsinstitut für Molekulare Pharmakologie (FMP), 13125 Berlin, Germany
[2]Deutsches Krebsforschungszentrum (DKFZ), Division of Translational Molecular Imaging, 69120 Heidelberg, Germany
[3]NMR Research Unit, University of Oulu, 90014 Oulu, Finland
[4]Research Unit of Medical Imaging, Physics and Technology (MIPT), University of Oulu, 90014 Oulu, Finland
[5]University of Jyvaskyla, Department of Chemistry, 40014 Jyväskylä, Finland
[*]Corresponding authors: perttu.lantto@oulu.fi, lschroeder@fmp-berlin.de

Contents

1 Synthetic scheme of Fe-MOP **1**

2 Structural characterization of Fe-MOP **1**

3 Fe-MOP stability under variable temperatures **3**
3.1 Stability under moderate heating 3
3.2 CEST responses at lower temperatures 3

4 Fe-MOP stability under different perturbation conditions **4**
4.1 Stability under O_2 atmosphere 4
4.2 Influence of excess impurity on Fe-MOP hyper-CEST performance 4

5 Comparison of hyper-CEST responses of fresh and stored Fe-MOP solutions **5**

6 Resolving the racemic mixture of Fe-MOP using (*S*)-BINOL **6**

7 Blocking the tetrahedral face of Fe-MOP using Gu·HCl **8**

8 Competition between Xe and larger guests for Fe-MOP cavity **8**
8.1 Cyclohexane and Xe competition 8
8.2 Competition between SF_6 and Xe 8

9 Impact of Xe bubbling on supramolecular Fe-MOP structure **8**

10 Direct HP Xe NMR spectra of Fe-MOP at different concentrations **10**
10.1 Inversion Recovery measurements 11
10.2 CEST responses of different pools at higher Fe-MOP concentration 12

11 Thermal CEST measurement **13**

12 CEST simulation for Fe-MOP sample at 25 °C **14**

13 First principles computational methods and results **16**
13.1 Conformational search of three Fe-MOP diastereomers 16
13.2 ^{129}Xe NMR chemical shifts in GFN2-xTB conformations 19
13.3 Thermal averages of ^{129}Xe chemical shifts in the lowest G conformations . . . 21

14 ^{1}H NMR spectrum of Fe-MOP with extended scans **23**

15 DFT/B97-3c/COSMO geometries **24**

Supplementary References **27**

1 Synthetic scheme of Fe-MOP

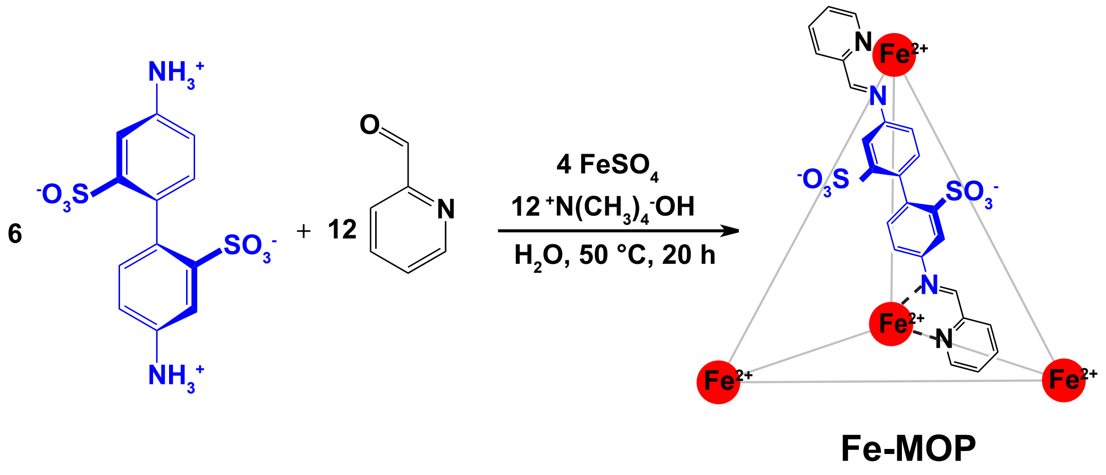

Supplementary Figure 1. Synthesis pathway of tetrahedral (T_d) Fe-MOP starting from 4,4'-diaminobiphenyl-2,2'-disulfonic acid and 2-formylpyridine in presence of iron (II) salt and excess tetramethylammonium hydroxide. For clarity purpose only one of the ligands oriented along the T_d edge for complexing two Fe^{2+} cations is shown.

2 Structural characterization of Fe-MOP

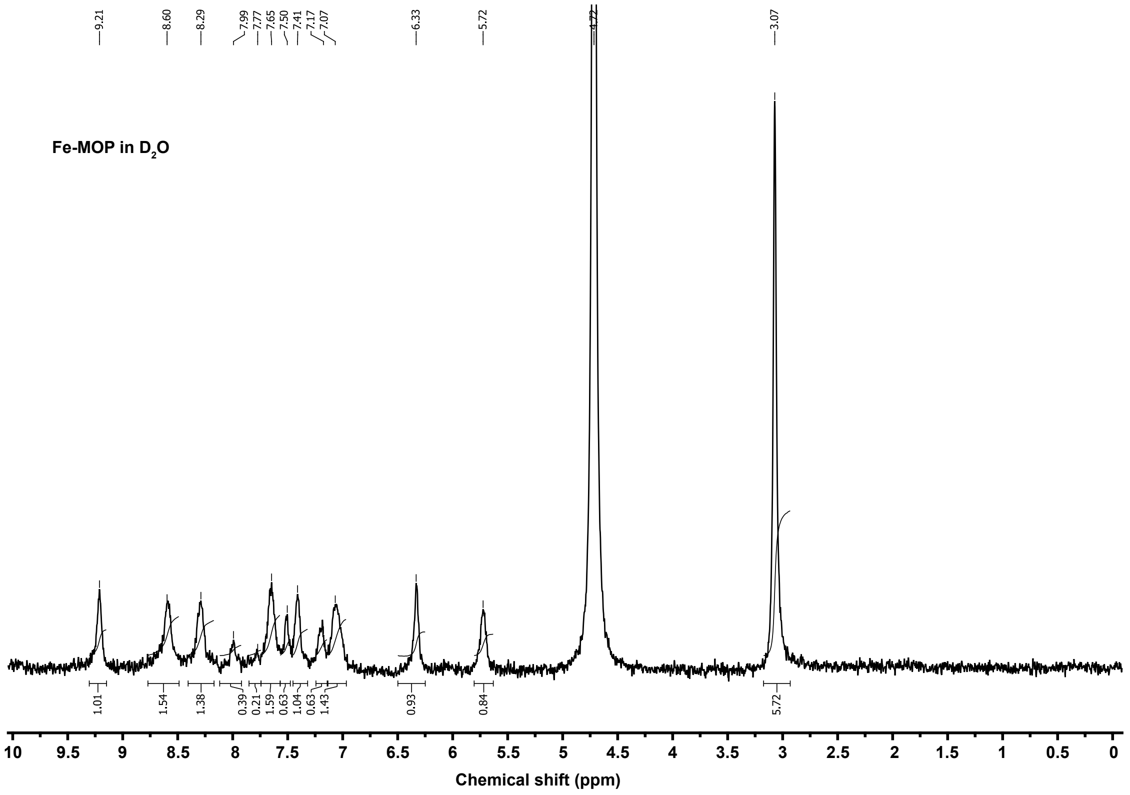

Supplementary Figure 2. ^{1}H-NMR of Fe-MOP recorded in D_2O. The acetone impurity was removed by five cycles of lyophilization and confirmed by lack of free and encapsulated acetone peaks (which would show up at 2.09 (free), 0.96 and 10 ppm (encapsulated), respectively). The observed peaks were in agreement with an earlier report [1].

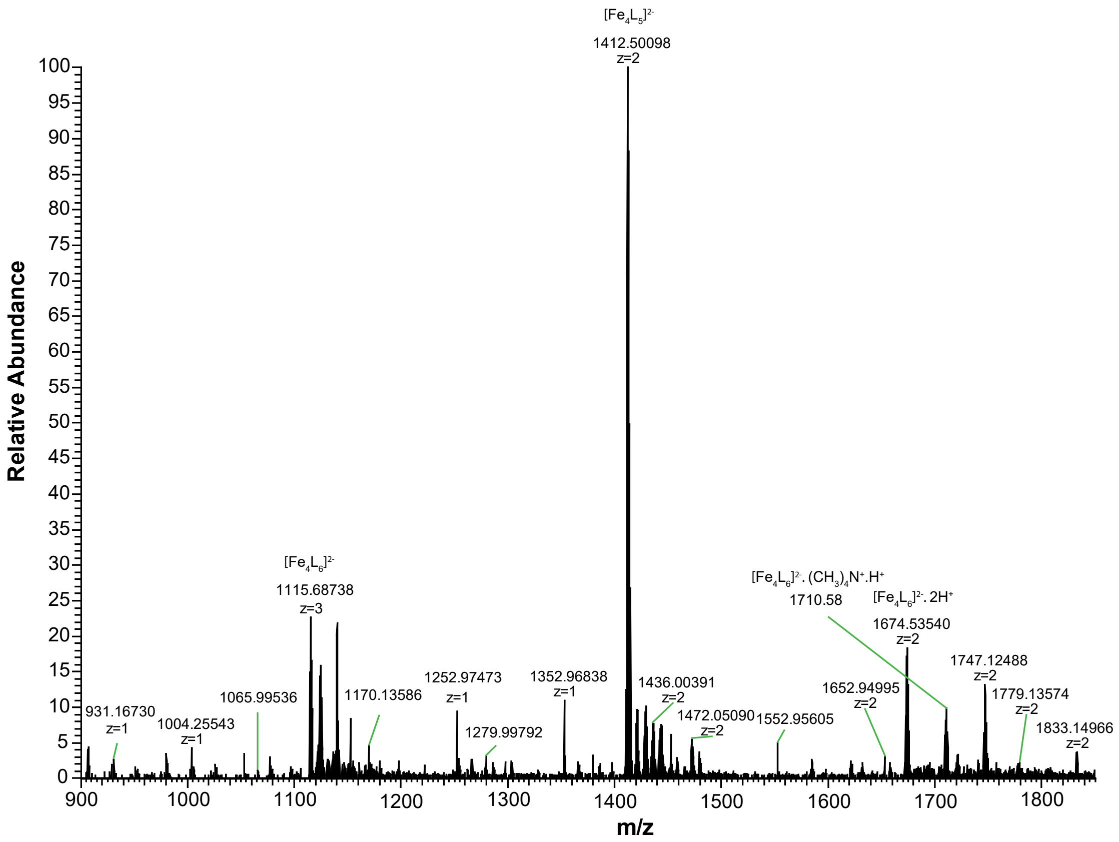

Supplementary Figure 3. ESI-MS spectrum of Fe-MOP measured in H_2O:MeOH mixture. The fragmentation pattern (m/z values) of Fe-MOP complies with its overall structure and is in agreement with the earlier report [2].

3 Fe-MOP stability under variable temperatures

3.1 Stability under moderate heating

Since the Fe-MOP is a self-assembled host system, testing its intactness and structural stability under different temperature conditions is of special interest. Different temperatures were achieved by Variable Temperature Unit (VTU) of the NMR spectrometer. Firstly, the Fe-MOP (100 μM) was measured at 25 °C by using the bubbling and saturation parameters described under the methods section of the main article. Consecutively, the temperature of Fe-MOP sample was increased in 5 °C increments using VTU from 25 °C up to 50 °C, followed by z-spectra acquisition at respective temperatures (see video in Supplementary Figure 4). Finally, Fe-MOP solution was cooled back to 25 °C for testing reproducibility and reversibility of Fe-MOP hyper-CEST responses. The results are shown in Figure 2 and discussed in the main article.

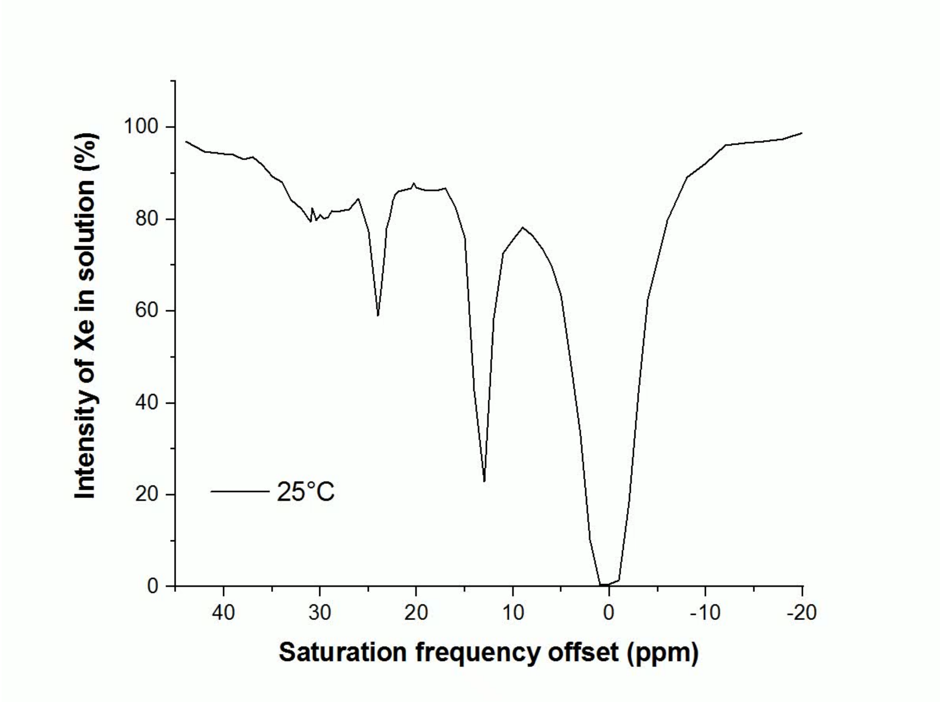

Supplementary Figure 4. First frame of an animated illustration of the changes in z-spectra for cycling through different sample temperatures. The full animation is available as Supplementary Video 1 on the journal's web page.

3.2 CEST responses at lower temperatures

The structural stability of Fe-MOP (100 μM) in H_2O was also checked at lower temperatures (4, 10 and 20 °C) by Xe hyper-CEST. Changes in the CEST responses are clearly visible and provide important additional information, particularly for the challenging fast exchange signal at ca. 30 ppm. (Supplementary Figure 5). While the other two signals mainly decrease due to decelerated and less efficient exchange, the broad signal at 30 ppm is increasing, getting narrower, and becoming more deshielded as temperature decreases and approaches the water density maximum (4 °C). At lower temperature the narrowing and increasing of the signal indicate slower but not restricted exchange. In fact, the deceleration makes the CEST effect more efficient. The residence time in the cage is extended and Xe probes more the cavity and, hence, signal moves closer to the chemical shift range of the Xe inside the (S_4) cavity. The intensities of the other two signals at 13 (T) and 24 ppm (C_3) decrease with the temperature, which indicates more restricted exchange between those Fe-MOP and water pools at low temperature.

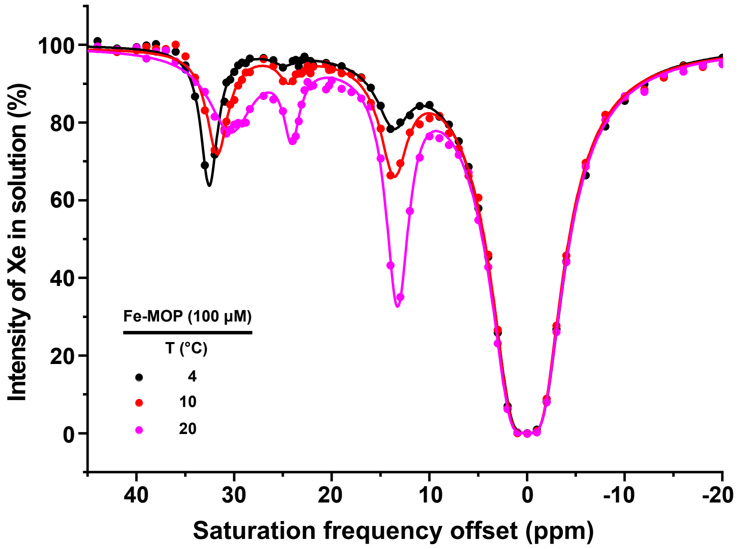

Supplementary Figure 5. The effect of low temperature on Fe-MOP encapsulation properties was studied by ^{129}Xe hyper-CEST. At lower temperatures (4, 10, 20 °C), three CEST peaks appeared at 13, 24 and 30 ppm, respectively. It appeared like the Xe exchange dynamics of CEST peaks at 13, 24 ppm differ from one at 30 ppm, because the 30 ppm peak is much broader and its position changes a lot with temperature.

4 Fe-MOP stability under different perturbation conditions

4.1 Stability under O_2 atmosphere

The stability of the coordinated Fe(II) in Fe-MOP was evaluated under O_2 atmosphere. This control study was carried out by bubbling pure O_2 for 10 min into the Fe-MOP (100 μM) for equilibration. Subsequently, Fe-MOP solution was maintained under O_2 atmosphere overnight. Then, the hyper-CEST response of the sample was measured and compared to Fe-MOP alone (Supplementary Figure 6).

4.2 Influence of excess impurity on Fe-MOP hyper-CEST performance

The influence of excess impurity on Fe-MOP hyper-CEST response was also investigated. It is known that the recrystallization solvent acetone can serve as a guest for Fe-MOP. Hence, the acetone-free Fe-MOP (100 μM) was intentionally mixed with excess acetone (150 mM) followed by immediate z spectrum acquisition at 25 °C (Supplementary Figure 6).

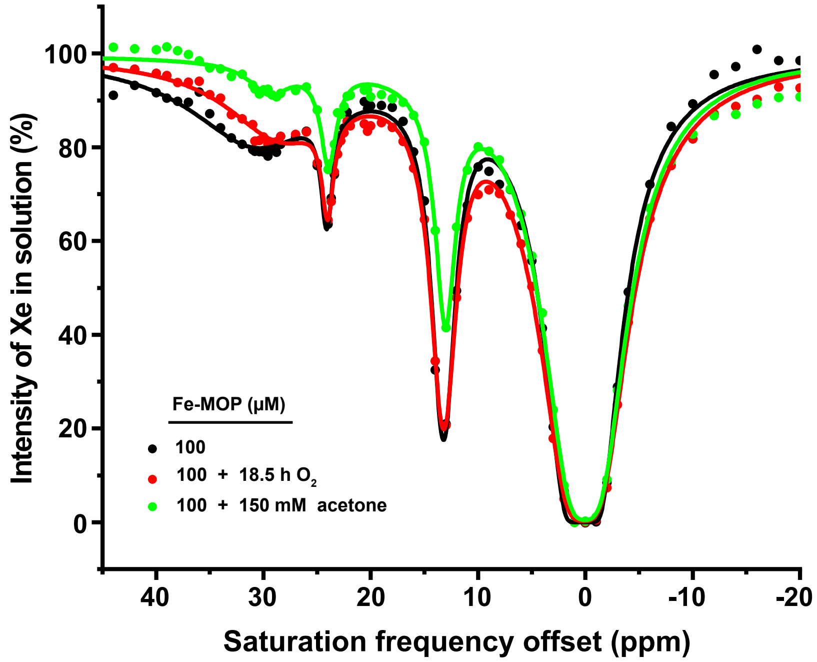

Supplementary Figure 6. Fe-MOP stability under different perturbation conditions. Fe-MOP treated with O_2 for 18.5 h resulted in a CEST spectrum similar to Fe-MOP alone. However, acetone (150 mM) encapsulated Fe-MOP indicated ca. 23% and ca. 10% reduction in 13 and 24 ppm CEST peak intensities compared to Fe-MOP alone.

5 Comparison of hyper-CEST responses of fresh and stored Fe-MOP solutions

The hyper-CEST response of the freshly prepared Fe-MOP solution is compared to that of stored solution in Supplementary Figure 7. The same hyper-CEST responses are immediately observed in the fresh solution.

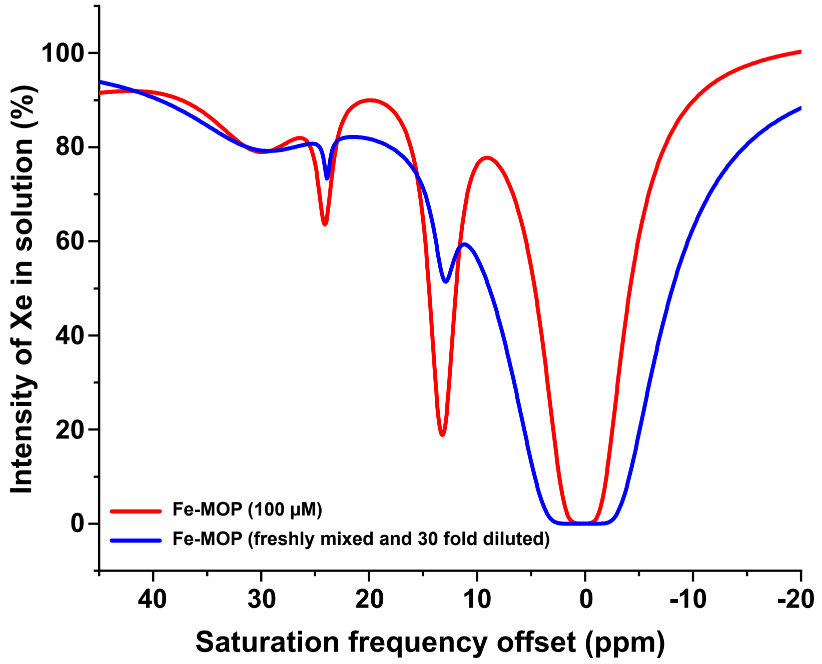

Supplementary Figure 7. z spectra clearly indicated that different population (diastereomers) of Fe-MOP is accessible since the commencement of the self-assembly. Measuring the reaction mixture (30-fold dilution i.e. ca. 267 μM) immediately after mixing the self-assembling organic components with iron(II) cations resulted in the blue spectrum. The z- spectrum (red) of final purified Fe-MOP (100 μM) is provided here as a reference.

The observed different signal intensities are presumably due to different exchange conditions. The direct saturation response at 0 ppm is rather broad for the fresh sample, thus indicating accelerated exchange kinetics until a full thermodynamic and kinetic equilibrium is reached. Since CEST is sensitive to the relation between exchange rates and applied saturation power, the faster exchange can make the saturation response appear reduced if the pulse power is kept constant. In this case, the CEST spectra of both fresh and stored Fe-MOP samples were acquired using similar saturation power (5.14 µT) applied for a length of 15 s. Furthermore, slightly different sample conditions such as residual base etc. after dilution can cause a pH change and thus impact the overall spectral response. Thus, it is not surprising that the signal intensities do not match since we are comparing incompletely formed Fe-MOP diastereomers to fully formed and purified counterparts. Since the reaction mixture was taken within ca. 5 min and diluted further, we propose as most likely explanation for the observed peak widths that the three diastereomeric cages of Fe-MOP have not yet formed completely to efficiently lock the incoming Xe guest. This is motivated by the observation that the direct saturation response around 0 ppm appears rather broad, thus indicating a frequent release from the pool of free Xe into the other pools. This pinpoints incomplete locking of the diastereomic cages of Fe-MOP (Supplementary Figure 7) in the case of freshly mixed solution after a very short reaction time (ca. 5 min) and might have led to the broadening of the signals. However, stored Fe-MOP solution prepared after 20 h reaction in water resulted in complete locking of diastereomic cages and subsequently, narrow CEST reponses for all three peaks. There is no structural difference observable between the freshly prepared and stored Fe-MOP in solid state (color and ^{1}H NMR) and in solution (ca. 4 months at 4 °C by color and ^{129}Xe HyperCEST NMR) that might have led to the observed difference in the CEST responses. This interpretation is also supported by practically unchanged chemical shifts.

6 Resolving the racemic mixture of Fe-MOP using (*S*)-BINOL

The racemic (*rac*) Fe-MOP was resolved by using (*S*)-BINOL according to the published protocol [2]. Briefly, *rac*-Fe-MOP (110 mg) and (S)-BINOL (38 mg) were added to 6 mL of H_2O:MeOH (v/v = 1:1) solvent mixture. The Fe-MOP was resolved by heating the reaction mixture at 50 °C for 1 h. After the heating, the less soluble diastereoisomer was collected by centrifugation and washed thrice with water (2 mL) and then once with acetone. Then the resulting residue was dried in vacuum and redissolved in 4 mL H_2O:MeOH (v/v = 1:1) solvent. The ΔΔΔΔ-isomer (D) was obtained from slow vapor diffusion of acetone into the H_2O:MeOH solution. The ΛΛΛΛ-isomer (L) was obtained by slow vapor diffusion of acetone into the filtrate. The successful separation of the *rac* mixture into D and L isomers was checked using CD spectroscopy.

The absorbance difference between the D and L forms in CD under the investigated concentration (250 μM) infer that the *rac* mixture contains an unequal structural content of these isomers due to an inefficient separation of two isomers. It appeared that the D isomer displayed a positive CD response higher in intensity than the negative CD response of L isomer. The negative CD response of L isomer remained unaffected in spite of increasing Fe-MOP concentration (Supplementary Figure 8). Increasing the concentration of L isomer compared to D isomer in Xe hyper-CEST experiments showed 3 CEST peaks with increasing peak intensities (Supplementary Figure 9).

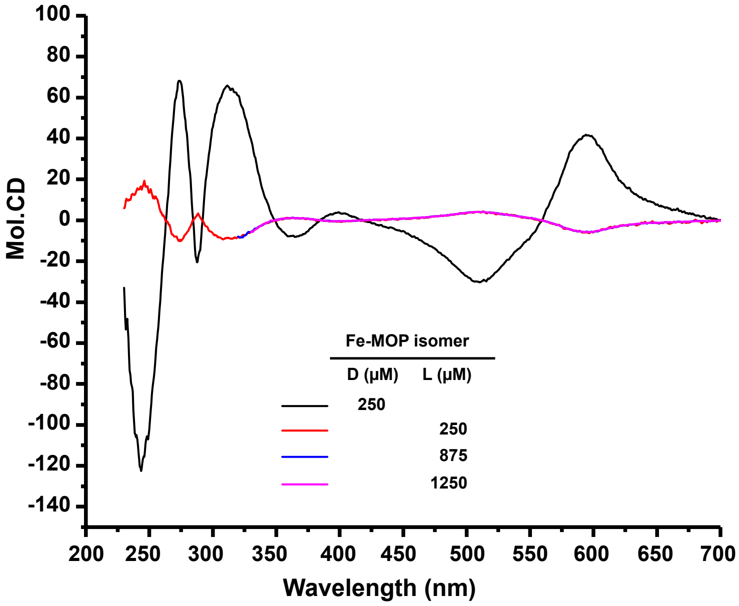

Supplementary Figure 8. CD spectrum of Fe-MOP D isomer (250 μM) showed intense peaks at 594, 510, 398, 311, 274 and 243 nm, respectively. The L isomer (250 μM) showed inverse CD response with reduced intensities. Increasing the concentration of L isomer (3.5 and 5 fold) matched the spectra of starting concentration (250 μM). This suggests that the observed CD signal strength difference between the D and L isomer could be attributed to incomplete separation of the *rac* Fe-MOP.

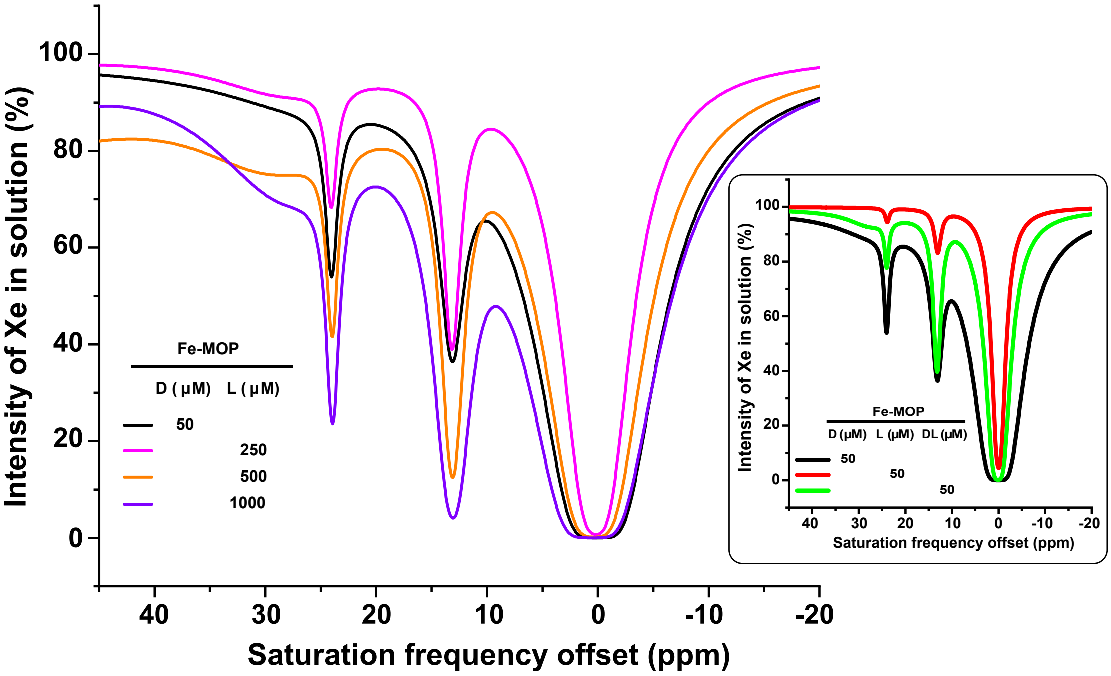

Supplementary Figure 9. The z spectra of D isomer (50 μM (inset)) showed 3 CEST responses similar to *rac*-Fe-MOP (50 μM). Conversely, L isomer (50 μM (inset)) shows strongly reduced signals with the fast exchange signal at ca. 30 ppm practically being undetectable and narrow Xe@solution peak (0 ppm). The D isomer hyper-CEST response was matched on reaching the following L isomer (~ 250 - 500 μM) concentration. However, *rac*-Fe-MOP (50 μM (inset)) displayed 3 CEST peaks and Xe@solution (0 ppm) resonance with peak width in between both isomers. This data confirms that both D and L isomer contribute to all three observed CEST signatures.

The difference in CEST signal intensities observed between D and L isomer of Fe-MOP is explained based on the inefficient separation of two isomers from *rac*-Fe-MOP. Based on the insights gained from CD spectroscopy and ^{129}Xe hyper-CEST, our interpretation is that the L isomer is not fully separated from its D counterpart. We have observed similar results as shown in Supplementary Fig. 9 despite repeating the optical separation multiple times. This led us to hypothesize that the *rac* mixture contains an unequal structural content of both D and L isomers.

7 Blocking the tetrahedral face of Fe-MOP using Gu·HCl

The Gu·HCl blocks the four tetrahedral (T_d) faces of Fe-MOP by initiating H-bonding [3]. The influence of Gu·HCl blockage on Xe encapsulation by Fe-MOP was tested by hyper-CEST. Fe-MOP (100 μM) was systematically blocked by gradual increase of Gu·HCl concentration (0.1 - 10 mM) followed by mixing and z spectra measurement (Figure 3, main article).

8 Competition between Xe and larger guests for Fe-MOP cavity

8.1 Cyclohexane and Xe competition

The selectivity of Fe-MOP for differently sized guests (e.g. Xe and cyclohexane (Cyhex)) at higher temperature (50 °C) was checked by ^{129}Xe hyper-CEST. In this study, Fe-MOP (100 μM) was mixed with Cyhex (15 mM) prior to bubbling HP Xe into the mixture and measuring it at 25 °C. The z-spectrum acquired at 25 °C served as a reference spectrum for no cyclohexane encapsulation. Then, the mixture was heated to 50 °C using the VTU unit. Subsequently, Xe was introduced during bubbling of the sample (Cyhex+Fe-MOP) and their competition for Fe-MOP cavity was followed at 50 °C. The time-resolved z-spectra of Fe-MOP in the presence of competing guests at 50 °C were recorded at 4, 8 and 24 h, respectively (Figure 4a, main article).

8.2 Competition between SF_6 and Xe

The influence of blocking the accessibility of Fe-MOP cavity to Xe by providing a larger competing guest was investigated by Xe-NMR. The Fe-MOP (100 μM) solution was equilibrated by bubbling SF_6 for 5 min. Subsequently, the Fe-MOP sample was maintained under SF_6 atmosphere for 3 days at 50 °C for accelerating the SF_6 encapsulation. A z-spectrum of $SF_6 \subset$Fe-MOP was measured at 25 °C (Fig. 4b, main article).

9 Impact of Xe bubbling on supramolecular Fe-MOP structure

We hypothesized that Xe bubbling into Fe-MOP solution might have led to generation of 3 CEST peaks as a consequence of structural rearrangements induced by Xe. This hypothesis was tested by performing control CD measurements as shown in Supplementary Figure 10. No changes could be observed due to Xe bubbling. It should be noted that the CD measurement does not allow to maintain a defined Xe atmosphere on top of the solution and slow degassing might occur.

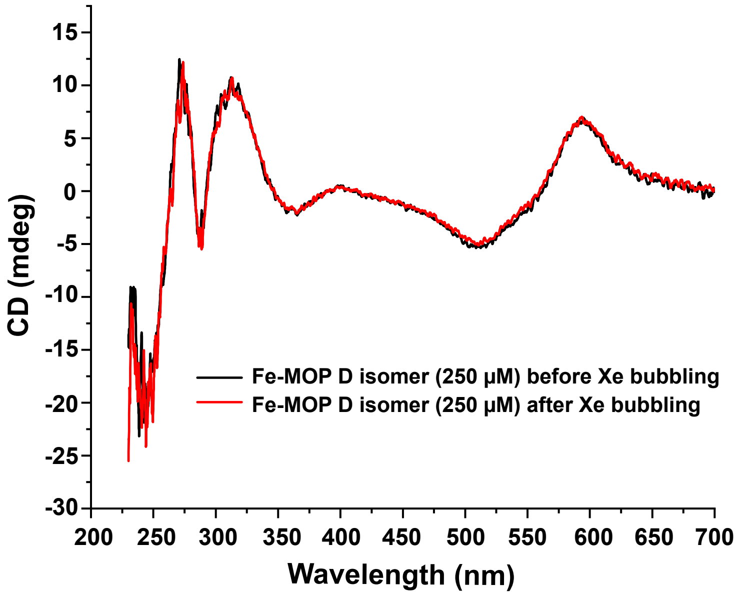

Supplementary Figure 10. The impact of Xe bubbling on delta (D) isomer of Fe-MOP structure was investigated by CD spectroscopy. Here, CD-responses of D isomer of Fe-MOP (250 μM) in H_2O was measured prior and after 15 min of Xe bubbling. The observed CD response indicates that no alteration occurred in Fe-MOP isomeric structure due to Xe bubbling.

10 Direct HP Xe NMR spectra of Fe-MOP at different concentrations

We also tried to detect different populations or 'hidden states' of Fe-MOP at different concentrations (10.3 mM and 100 μM) by direct HP ^{129}Xe-NMR. This experiment should rule out the speculation if the observed CEST peaks are occurring only as a hyper-CEST artifact. The direct HP ^{129}Xe-NMR revealed a solution peak and the two most prominent cage signals. The two bound Xe signals at 13 and 24 ppm were arbitrarily assigned as Cage1 and Cage2, respectively. The less intense Xe@solution (solution), an intense Xe@Fe-MOP (Cage1) and a very low intense Xe@Fe-MOP (Cage2) peaks are observed in Supplementary Figure 11a due to the usage of higher host (10.3 mM) and increased Xe concentrations (5 % for line narrowing). The higher Xe host concentration and the lower temperature (4 °C) for line narrowing enabled us for the first time to detect sparsely populated Fe-MOP at 24 and 30 ppm in conventional direct Xe spectra (Supplementary Figure 11a,b). The data acquired at 4 °C was analyzed by fitting Lorentzian line shapes to yield qualitative information about different pool sizes (Supplementary Figure 11c). Conversely, employing lower Xe (2 %) and host concentration (100 μM) revealed an intense Xe@solution (solution) and low intense Xe@Fe-MOP (Cage1) peaks (Supplementary Figure 11d). In spite of increasing the number of scans and lowering the temperature of the sample, we could observe only two peaks in Supplementary Figure 11d similar to our earlier thermally polarized Xe spectra. [4]

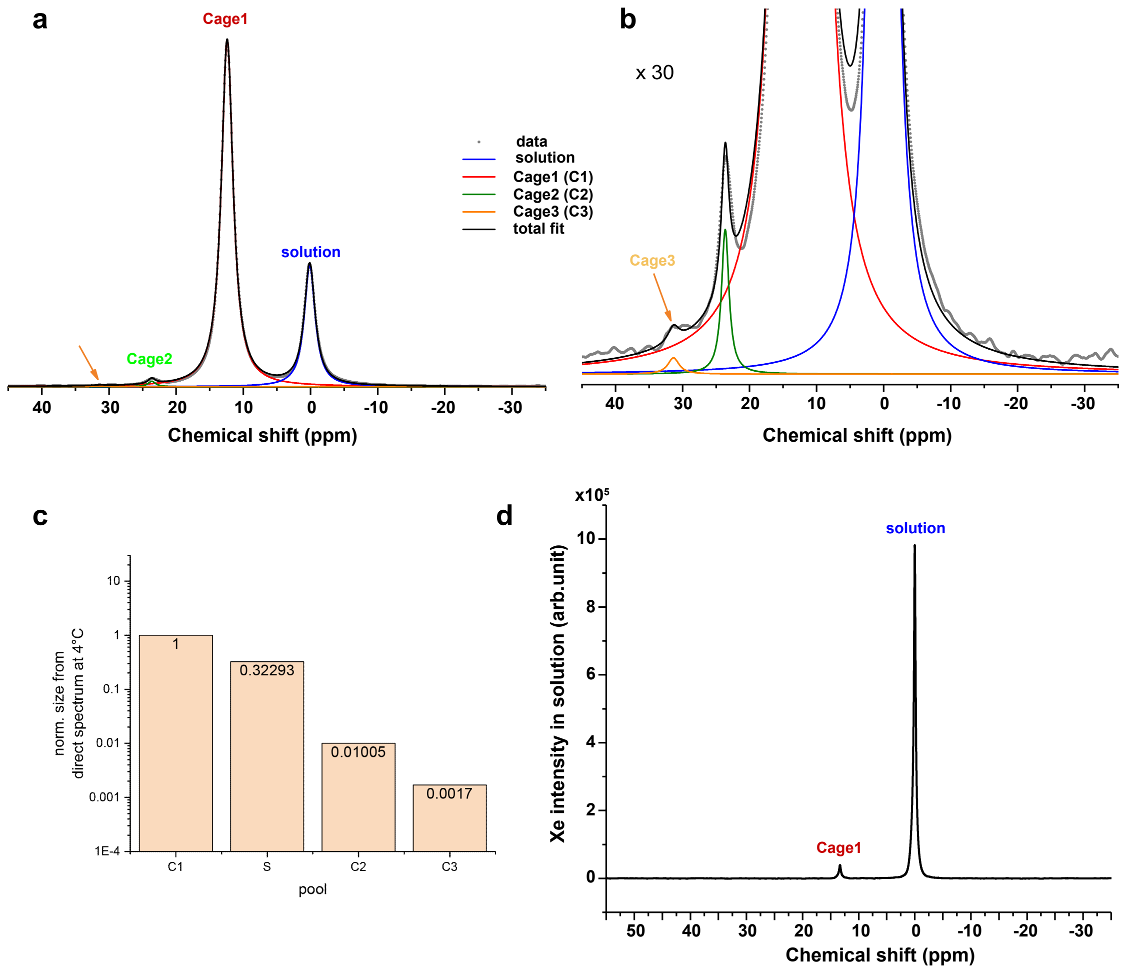

Supplementary Figure 11. Direct HP Xe-NMR of Fe-MOP (10.3 mM (a,b) and 100 μM (d)) recorded in H_2O. a: Owing to higher Fe-MOP concentration and 5 % hyperpolarized Xe, an intense Xe bound peak (Cage1) exceeding the solution peaks is seen. Here, for the first time, a second very low intense and broad bound Xe peak (Cage2) at 24 ppm (a,b) was observed in addition to 30 ppm peak (Cage3) that was made visible by applying line broadening ($\sim$ 100 Hz) and 30 fold magnification on the spectra in (a). c: Fitting Lorentzian line shapes to the direct spectra acquired at 4 °C revealed the pool sizes qualitatively. d: Direct ^{129}Xe-NMR of Fe-MOP (100 μM) revealed an intense solution and low intense Cage1 peaks similar to spectra acquired using thermally polarized Xe.

10.1 Inversion Recovery measurements

A representative direct Xe NMR spectrum utilized for inversion recovery measurements is shown in Supplementary Figure 12. To understand the interlink between different pools, the two bound Xe peaks and Xe@solution are arbitrarily assigned as Cage1, Cage2 and solution peaks. Direct Xe NMR of Fe-MOP (10.3 mM) acquired at 5 % Xe showed different peaks with intensities of Cage2 (1.23 %) Cage1 (73.79 %) Solution (24.98 %), respectively, yield the total intensity to be 100 %. The line widths for Cage2, Cage1 and solution are 145 Hz, 150 Hz and 107 Hz, respectively.

We performed inversion recovery (IR) measurements by using a modified selective Gauss inversion pulse with 500 Hz bandwidth and a duration of 1064 μs followed by a block pulse read out. The frequency offset for the inversion pulse was referenced to the intense cage peak (Cage1) and 1094 data points of the FID were filled up to 2048. A line broadening of 20 Hz was utilized for this IR measurement and 16 scans were performed for each inversion time (TI). The used TI values are 1, 1, 10, 20, 50, 100, 150, 200, 250, 300 and 350 ms, respectively. The first TI setting was used as a dummy scan.

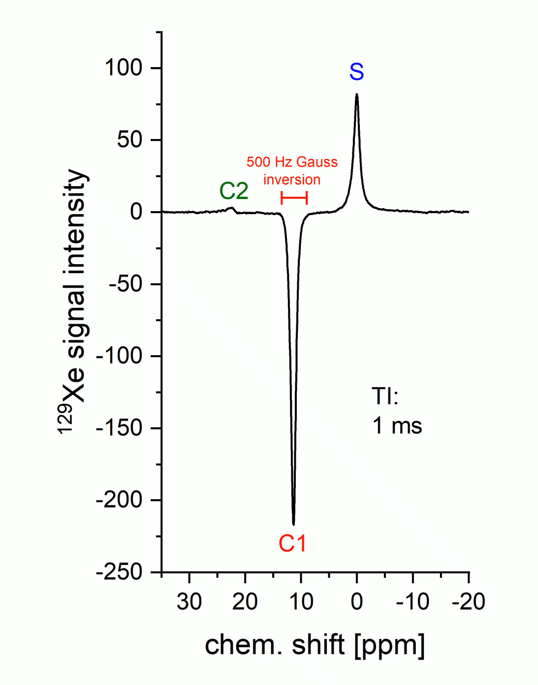

Supplementary Figure 12. First frame of a video showing the selective inversion of one of the three peaks while monitoring the overall signal changes over 350 ms. The full animation is available as Supplementary Video 2 on the journal's web page.

The three site exchange model used for fitting to obtain exchange rates from the time-dependent signal intensities in the spectra is shown below.

$$\text{Cage1} \underset{k_{12}}{\overset{k_{21}}{\rightleftharpoons}} \text{Solution} \underset{k_{23}}{\overset{k_{32}}{\rightleftharpoons}} \text{Cage2}$$

According to the fits, the exchange rates between the solution and the Cage1 sites are $k_{12} = 8.8 \pm 0.6$ s^{-1} and $k_{21} = 2.98 \pm 0.11$ s^{-1}. The exchange rates for the Cage2 pool have large error margins due to very small amplitude of the signal. In the best fit, the values were $k_{23} = 20 \pm 10$ s^{-1} and $k_{32} = 0.8 \pm 0.4$ s^{-1}. Note that these values differ from the values given in Table S2 as the concentration of cages in the IR experiments (10.3 mM) was much higher than in the CEST experiments (100 μM).

10.2 CEST responses of different pools at higher Fe-MOP concentration

In this experiment, Fe-MOP (10.5 mM) in H_2O was bubbled with a gas mixture of 5 % Xe, 10 % N_2 and 85 % He for 10 s, followed by a waiting time of 6 s for foam to disappear. The CEST measurements were performed at two different temperatures (10 and 25 °C). Similar to IR measurements, the Xe@solution, intense Xe@Fe-MOP peak, and low intense Xe@Fe-MOP peaks are termed solution, Cage1 and Cage2 pool, respectively (Supplementary Figure 11a). A 100 Hz line broadening was used in order to reduce the noise level particularly for cage2 peak.

The CEST measurements at 25 °C were carried out by applying saturation pulses (block pulse) with step size of 1 ppm within 30 to -30 ppm saturation frequency offsets (16 acquisitions) followed by observing the change in the intensity of different CEST pools (Solution, Cage1, Cage2, Cage3). The saturation strength of B_1 = 2 μT was applied for a length (t_{sat}) of 250 ms.

The CEST measurements at 10 °C were performed under similar experimental conditions as described above, except the saturation of different pools were performed using a higher strength of B_1 = 5 μT at unchanged length (t_{sat} = 250 ms).

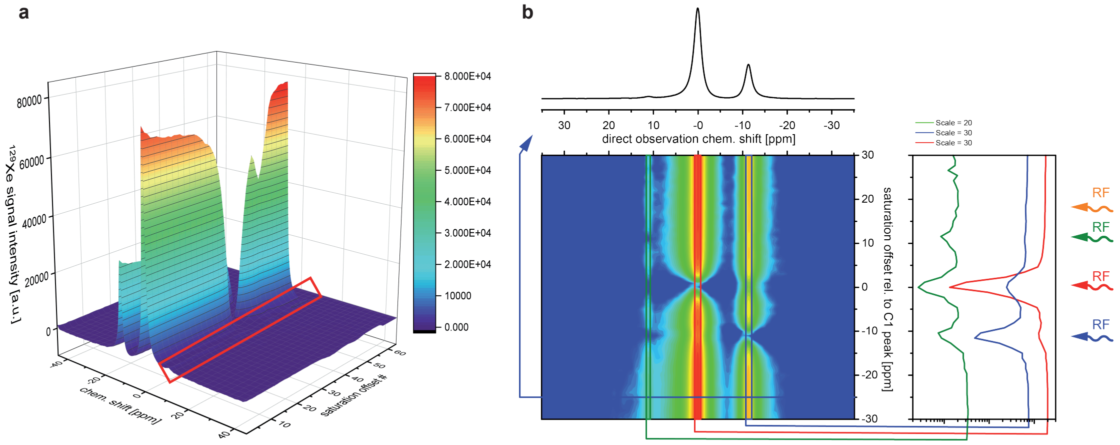

Supplementary Figure 13. 2D CEST data of Fe-MOP (10.5 mM) recorded in H_2O at 25 °C. (a): The color coded plot of 2D CEST data clearly shows the various responses from different CEST pools (solution, Cage1, Cage2). The Cage2 peak is visible as a shoulder (see red box) next to the intense Cage1 peak. (b): Plotting the 2D CEST data on a logarithmic scale showed responses with different intensities. The qualitative CEST response of different peaks upon saturating the different signals is shown on the right.

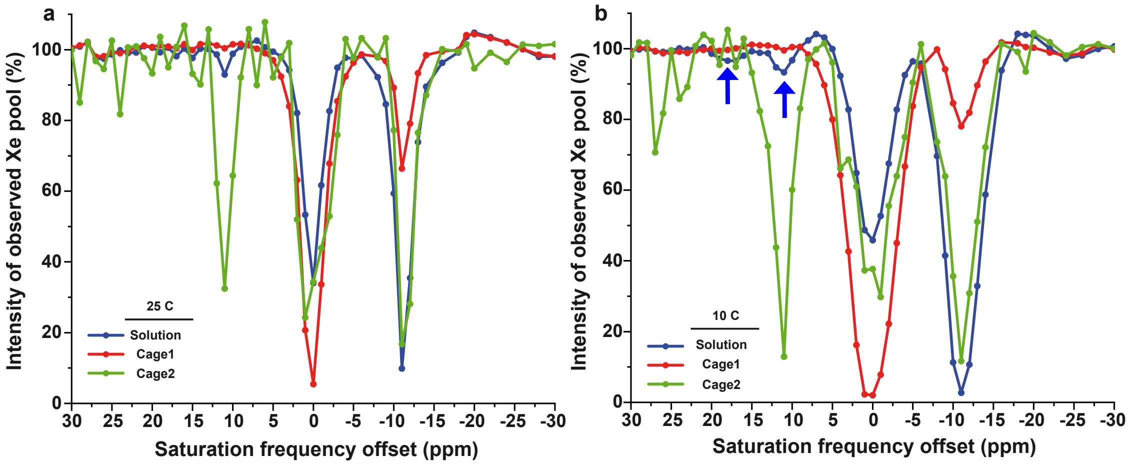

Supplementary Figure 14. CEST intensities of observed Xe pools of Fe-MOP (10.5 mM) at 25 (a) and 10 °C (b). (a): Saturating different peaks (Solution, Cage1, Cage2) at 25 °C showed CEST peaks with different intensities. (b): Cooling the Fe-MOP solution to 10 °C reveals that all 4 CEST signals can only be detected in the solution peak after a 250 ms saturation. Thus, only this pool picks up immediate exchange with all other pools.

It is noteworthy that the CEST spectrum of Cage2 does not necessarily react best to direct saturation. Due to the relative pool sizes, spins in Cage2 have a rather short residence time, such that direct saturation is relative inefficient (the situation improves a little for cooling down to 10 °C). Indirect saturation is better as the solution pool and the Cage1 pool are more "receptive" due to their longer residence time. The effectiveness of their "input" into Cage2 in then determined by their pool size and connectivity: Cage1 is larger and can accumulate quite some saturated magnetization but it is only indirectly linked to Cage2. The smaller solution pool is also receptive for saturation but can directly feed into Cage2.

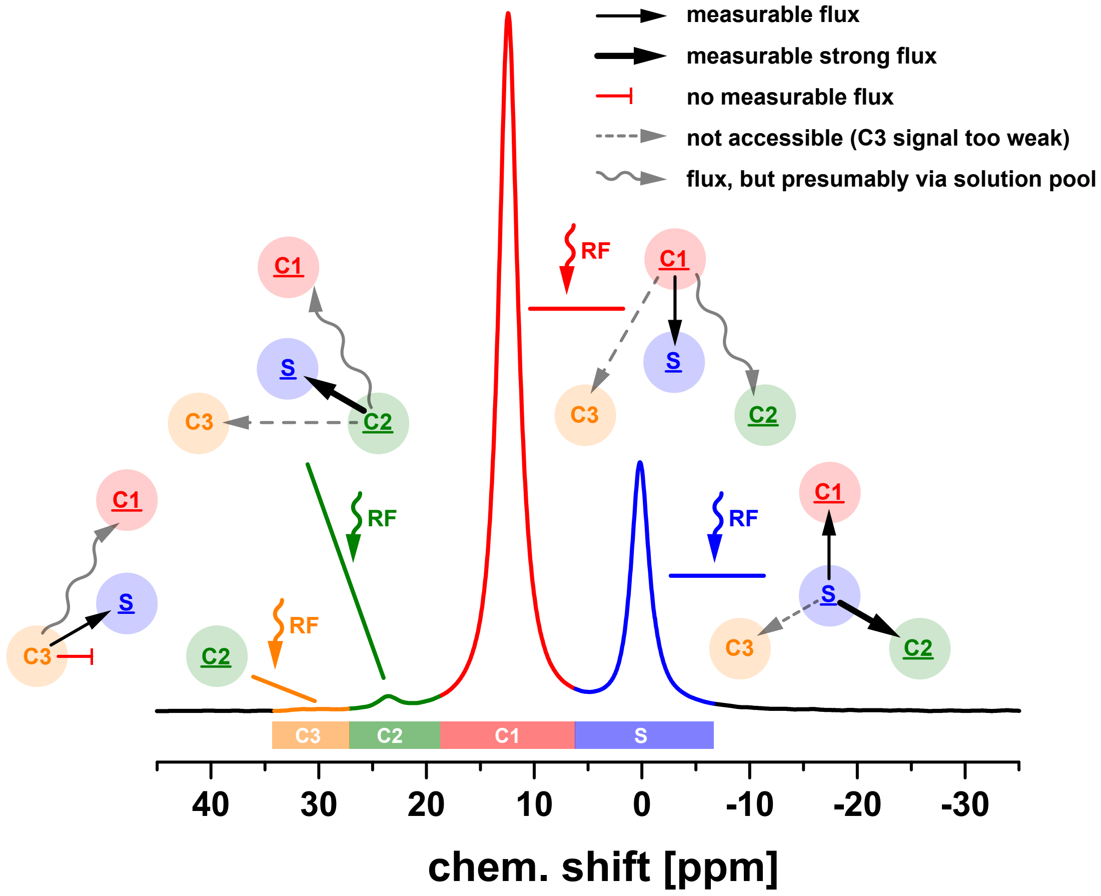

Supplementary Figure 15. Possible exchange network between different CEST pools. For example, saturating the solution pool indicated a measurable strong Xe exchange flux in Cage2 compared to Cage1. The flux network between Cage3 and other pools are not accessible as it might occur at even shorter time scale thus making it invisible for CEST detection. However, saturating one of the cage pools (Cage1, Cage2, Cage3) showed an immediate measurable strong flux in the solution pool. The flux between Cage1, Cage2 and Cage3 is proposed to occur presumably via the solution pool.

11 Thermal CEST measurement

Thermal CEST spectrum of Fe-MOP (8.9 mM) was recorded within the chemical shift range of 165 to 260 ppm by performing 64 scans using Xe at 4.9 mbar pressure. The sample was irradiated with 30 mW power for 2 s. Thermal CEST spectrum clearly showed the presence of Cage3 signal at 30 ppm which remained undetected even after recording the thermal ^{129}Xe spectrum with long acquisitions (10240 scans). The thermal CEST spectrum is shown below (Supplementary Figure 16) and it also confirmed the presence of three diastereomers of Fe-MOP similar to Xe hyper-CEST spectrum.

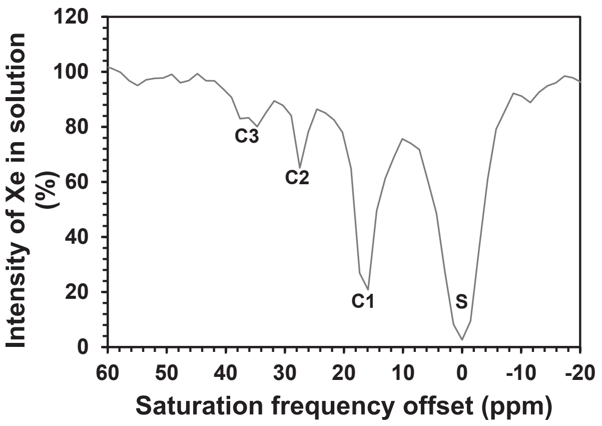

Supplementary Figure 16. Thermal CEST spectrum of Fe-MOP (8.9 mM) recorded using 30 mW and 2 s irradiation.

12 CEST simulation for Fe-MOP sample at 25 °C

We have performed the simulation of CEST data as an approach to fit the z-spectrum obtained at 25 °C by utilizing the four-site exchange model and subsequently estimated the populations of different exchange sites available in Fe-MOP (Supplementary Figure 17 and 18).

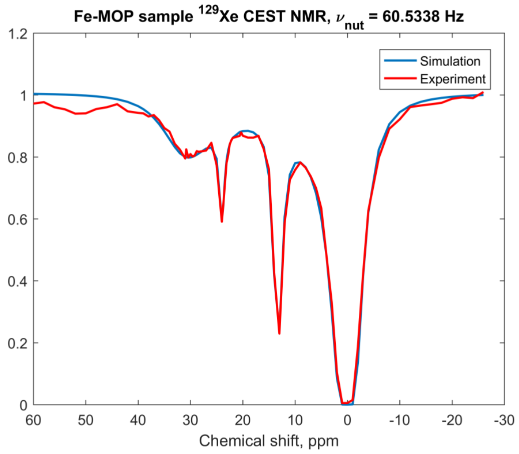

Supplementary Figure 17. CEST simulation result (blue) and the experimental data (red). The parameters used in the simulation are shown in the table below

The simulation of the CEST experiment was carried out by using four sets of Bloch's equations [5] connected by kinetic terms according to Supplementary Figure 18.

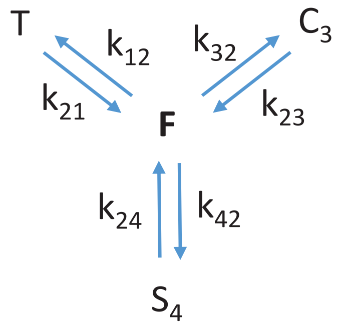

Supplementary Figure 18. Four-site exchange model used in the simulation of the CEST experiment

The resulting set of equations for different resonance offsets linked to the z spectra were solved numerically using MATLAB. The z-spectrum represents the normalized ^{129}Xe NMR

signal amplitude of unbound Xe in Supplementary Figure 16 as a function of the RF resonance offset.

Some required parameter estimates for the simulation were derived from the supplementary inversion recovery measurements, direct ^{129}Xe NMR spectra and literature data [4] (k_{12}/k_{21} ratio; chemical shifts of "F", "T", "C_3" and "S_4"; k_{21} and k_{23} estimates; T_2 and T_1 time estimates), respectively. The exchange rate constants and the relaxation times were optimized through several iterations in order to achieve a good fit of the experimental points. Nutation frequency (ca. 60.53 Hz) required for simulation was determined from the experimental B_1 field amplitude (5.14 μT). The optimized exchange constants were used to calculate the populations of each sites. Different parameters utilized for fitting are shown in the Supplementary Tables 1 and 2.

Supplementary Table 1 Relaxation times, chemical shifts and site populations from the CEST simulation

Site	T_1, s	T_2, s	δ, ppm	Population
S_4	11	1	31.5	4.1 x 10^{-4}
C_3	11	1	24	1.4 x 10^{-3}
T	11	0.5	13.3	3.8 x 10^{-2}
F	60	2	0	9.6 x 10^{-1}

Supplementary Table 2 Exchange rate constants used in the CEST simulation

Exchange rate constant	k, s^{-1}
k_{12}	1.08 x 10^{-1}
k_{21}	2.7
k_{32}	2.7 x 10^{-2}
k_{23}	1.8 x 10^{1}
k_{42}	1.7
k_{24}	4.0 x 10^{3}

Subjecting the conventional ^{129}Xe NMR to calculations using the parameters obtained from the CEST simulation revealed a good agreement with the experimentally acquired spectrum (Supplementary Figure 19)

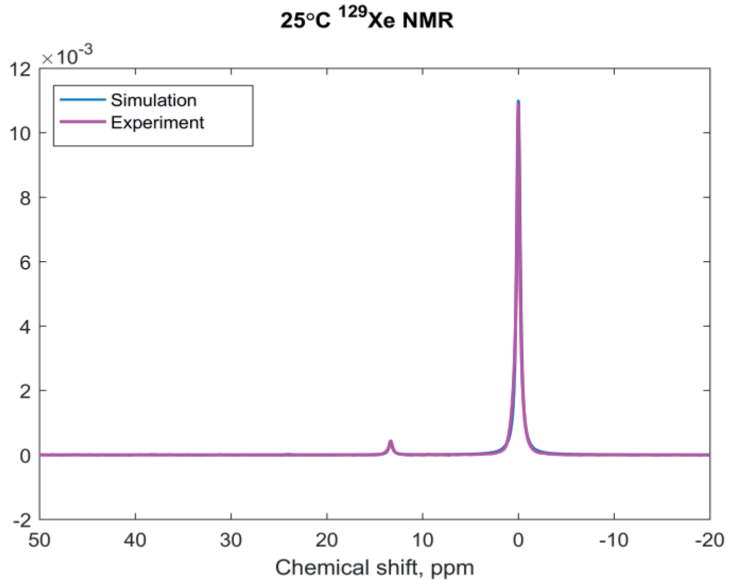

Supplementary Figure 19. Direct ^{129}Xe NMR spectrum simulated with the parameters from the CEST simulation (blue) and the experimental spectrum (magenta).

13 First principles computational methods and results

13.1 Conformational search of three Fe-MOP diastereomers

Motivation In order to obtain the likely structure of the three main isomers, it was necessary to come up with a heuristic method incorporating some prior knowledge on top of a systematic conformational search. This is for several reasons. 1) the energy barriers between possible conformers are too high (not accessible by room-temperature simulation, see below paragraph"Energy barriers") at the same time, an accurate forcefield parameters for Fe(II)-N bonds are not available to employ reliably a simulated annealing or other low-energy-structure-search algorithm. 2) more importantly, the structure is critically dependent on accurate solvation energy (free energy) and related hydrophobic effect with the important entropic contribution of the solvent. It is therefore practically impossible to evaluate the accurate free energies of the system for an order of 10^9 of rotamers/conformers. The ranking of structures has to be based on single-point energy, possibly with fast-to-evaluate entropic term of the solute only. Prior knowledge is therefore needed to filter-out structures that would pass the energy criterion, but which have unrealistic solvation situation, in our case, buried hydrophilic moieties.

The other option could be to use or develop a tool with a prior knowledge about solvation situation already incorporated. Such an idea reduces to maximization of the hydrophobic packing in a highly successful protein-fold search engine Rosetta and its variants (see e.g. Ref. [6]), where all the charge-charge interactions are by default plainly discarded. Here, we kept all the atomic interactions during the energy minimisation and ranking, which required the consequences of the hydrophobic character of the cavity and the Xe atom interactions to be addressed as the filter for the conformers.

Modeling method In our previous modeling of Fe-MOP cage with Xe, [4] the structure was deduced from X-ray data and only the positions of four counter ions (NH_4^+) on the faces of the cage were optimized. To cover the conformational space of the different stereoisomers, it is necessary to generate conformers inaccessible from one local minimum by low-energy motions. We refrained from simply exchanging the inclination of the SO_3 groups on the linker, albeit more starting points were generated for subsequent energy minimization. To systematically search for the possible conformers of each diastereomer, all the benzylsulphonate groups were rotated in 5 steps per full rotation. For 12 benzylsulphonate groups, this procedure resulted in $5^{12} \sim 0.24 \times 10^9$ conformers.

Unrealistic conformers were then filtered as described below in paragraph “Filters”. The remaining structures were subjected to minimal solvation i.e. described below in paragraph “Minimal solvation”. Xe atom was placed into the solvated and neutralized cages and optimized using the semiempirical extended tight binding GFN2-xTB method [7] via the XTB code [8]. Additionally, generalized Born surface area (GBSA) [9] implicit solvated model with parameters for water was used along with the explicit solvation.

Unrealistic conformers were again filtered as described below in paragraph “Filters”. The free energy estimates of remaining few (≤ 20) conformers of each diastereomers were achieved by subjecting the lowest GFN2-xTB energy (including only solvation free energy estimate) to vibrational analysis calculation on XTB code. By default, the XTB program discards the low-frequency modes, therefore the vibration of the explicit solvent molecules in the solute potential do not cause complications. The Xe NMR nuclear shielding was calculated for above conformers without further geometry optimization.

Minimal solvation Each oxygen atom in SO_3 group was solvated by one water molecule only, thus generating 36 water molecules for 12 SO_3 groups. Each water molecule was oriented along the S-O axis. It was further iteratively adjusted through rotation around its axis and around a cone defined by the S-O axis in order to avoid overlaps from van der Waals diameters. To compensate the overall charge of the complex, four ammonium ions were placed above the centers of the four walls of the approximately tetrahedral structure. Further, the positions of these ions were adjusted iteratively to avoid overlaps within van der Waals radii. The unrealistic structures were further filtered if any atom of SO_3 groups, and oxygen or nitrogen atoms from solvating H_2O or NH_4 ions enters the approximate tetrahedron defined by the four Fe atoms.

Filters The first filter removed structures with overlapping vdW radii of atoms. The second filter removed structures with hydrophylic sulphonate groups inside the tetrahedron formed by the Fe atoms (see Supplementary Figure 20). The third filter was only applied for the optimized structures and it removed structures with Xe outside the cage and/or sulphate groups below the purple plane shown in Supplementary Figure 20.

Energy barriers While generating possible conformers for a given stereoisomer, it is helpful to estimate the energy barrier for exchanging sides of the SO_3 groups as it serves as an easy step for conversion between different low-energy conformers. We used the push-pull reaction path estimate algorithm of the XTB program [8] to calculate this energy barrier for a T diastereomer without including any solvation or counter-ions. Since the steric limitation experienced during the transition requires bending of several bonds, excluding the stabilizing solvent and counterions should result in reasonable first approximation. We found a barrier of 68 kcal/mol as the lowest estimate, inaccessible at room temperature. The conversion between different conformers would not be feasible only through this simple step.

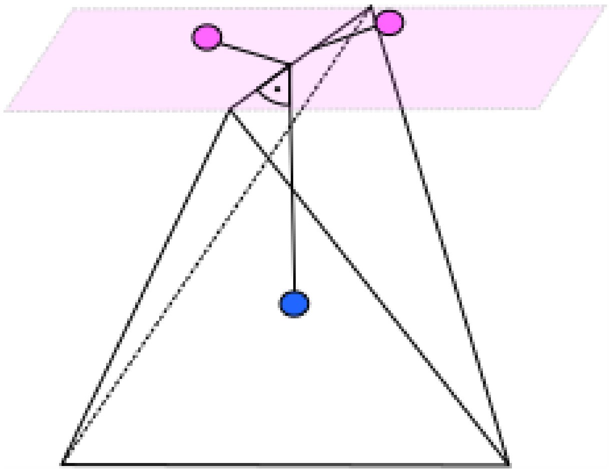

Supplementary Figure 20. Scheme of a plane that served as the final filter for the accepted structures. Structures with one or more SO_3 groups (depicted as magenta balls) below the plane were discarded. The blue ball shows the center of the tetrahedron defined by the four Fe(II) atoms.

RMSD analysis of conformers We have calculated root-mean-square-distance (RMSD) between conformers of each stereoisomer to assess if the generated conformers, after rounds of geometry optimisation, remained sufficiently distinct, or if some converged into clusters of very similar minima.

Due to possible symmetry of the structures, it is not sufficient to calculate RMSD without probing the distance also for all symmetry-related structures. This can be rigorously done by considering symmetry of the molecular graphs [10]. For our molecules, such algorithm is computationally too heavy, so instead, we implemented all 24 symmetries of the tetrahedral symmetry to create all the symmetry images before calculating the RMSD and selecting the minimum as the final value. Because after symmetry operations the atoms with the same indices are not anymore those that should be compared, the RMSD was calculated using "Hungarian algorithm" [10–12], which is fast and sufficient algorithm for our problem.

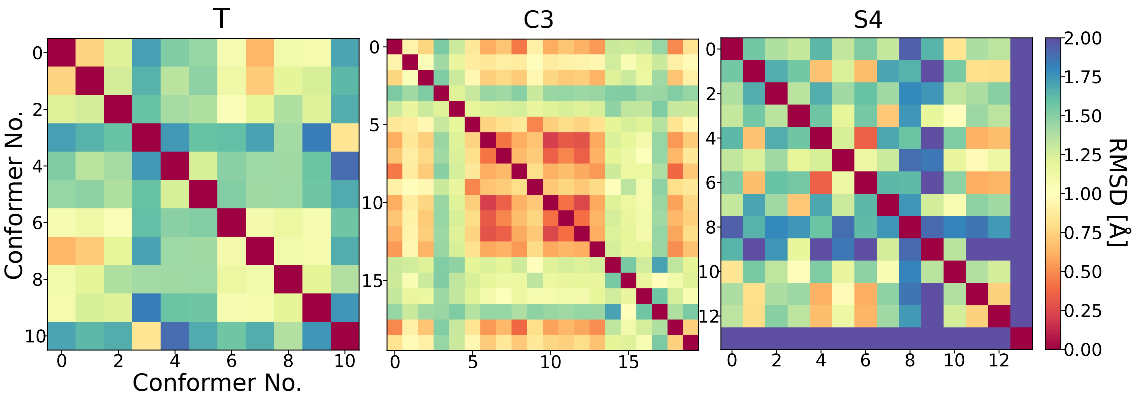

Supplementary Figure 21. Matrices of RMS distances between all low-energy conformers listed in Table 3.

The RMSD results, with numbering of conformations corresponding to Supplementary Table 3, are shown at Supplementary Figure 22. There are indeed clusters of structures that have very similar local minima. For example, structures 6 and 10 of C_3 stereoisomer have RMSD of only 0.29 Å. Visual inspection confirms that these are practically the same structures. This shows that the initial set of structures was large enough, allowing more than one starting structure to reach the same minimum. Majority of the less than 20 chosen conformers are nevertheless distinct, such as arbitrary pair of conformers C_3 (number 1 and 11 in the matrix Fig. 21), with RMSD$= 0.92$ Å, shown in Supplementary Figure 23, where SO_3 groups have different orientations.

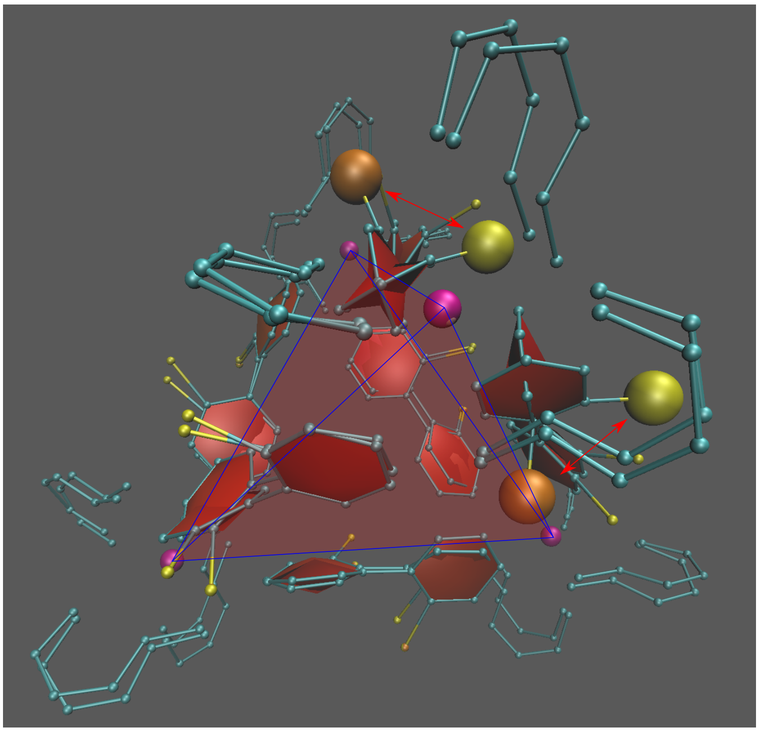

Supplementary Figure 22. Two conformers of C_3 stereoisomer, both in the principal frame of the tetrahedral symmetry defined by four Fe atoms, with mutual RMSD$= 0.92$ Å. Two sulphate groups have opposite orientations with respect to the corresponding linkers. This is shown as yellow (first conformer) and orange (second conformer) balls for the corresponding sulphur atoms. A subset of heavy atoms is shown to illustrate that most of them are close in space.

We have learnt from optimization of different conformers generated for the T diastereomer that the conformer in which the SO_3 groups orient itself similar to crystallographic structure displayed the lowest free energy conformation. Since the final conformation successfully passed different procedures such as several iterations of solvation with water molecules, counterions and optimizations resulting in conformation similar to the crystallographic structure, we are quite confident that the conformational search holds true for other stereoisomers, whose "real" conformation is unknown, and provides good preliminary

structures for them.

13.2 ^{129}Xe NMR chemical shifts in GFN2-xTB conformations

The calculated ^{129}Xe chemical shifts for the static above-mentioned optimized conformations of T, C_3, and S_4 diastereomers (see chosen computational level for NMR shielding in footnotes of Supplementary Table 3) are shown in Supplementary Table 3 and Figure 21. We do not observe the usual decreasing chemical shift trend with increasing cavity size [13, 14], since conformational changes of the ligands lead also to a higher variety of the cavity shapes (the mean volumes are 70, 63, and 68 Å^3 for T, C_3, and S_4, respectively; Xe van der Waals volume [15]: 44 Å^3). As the optimized (static) cavities are quite small, the Xe chemical shift (with respect to free Xe gas atom) becomes quite large. Together with the large variety of cavity shapes, we expect overestimated chemical shift ranges within and between the three diastereomers. The systematic errors are mainly due to approximate static equilibrium structures as well as neglecting the dynamics of Xe atom, cage, and solvent atoms. This inhibits quantitative treatment of ^{129}Xe NMR shifts inside Fe-MOPs [4, 16–18]. Instead, the obtained results provide reasonable preliminary estimates for the relative ranges of ^{129}Xe chemical shifts in different diastereomers.

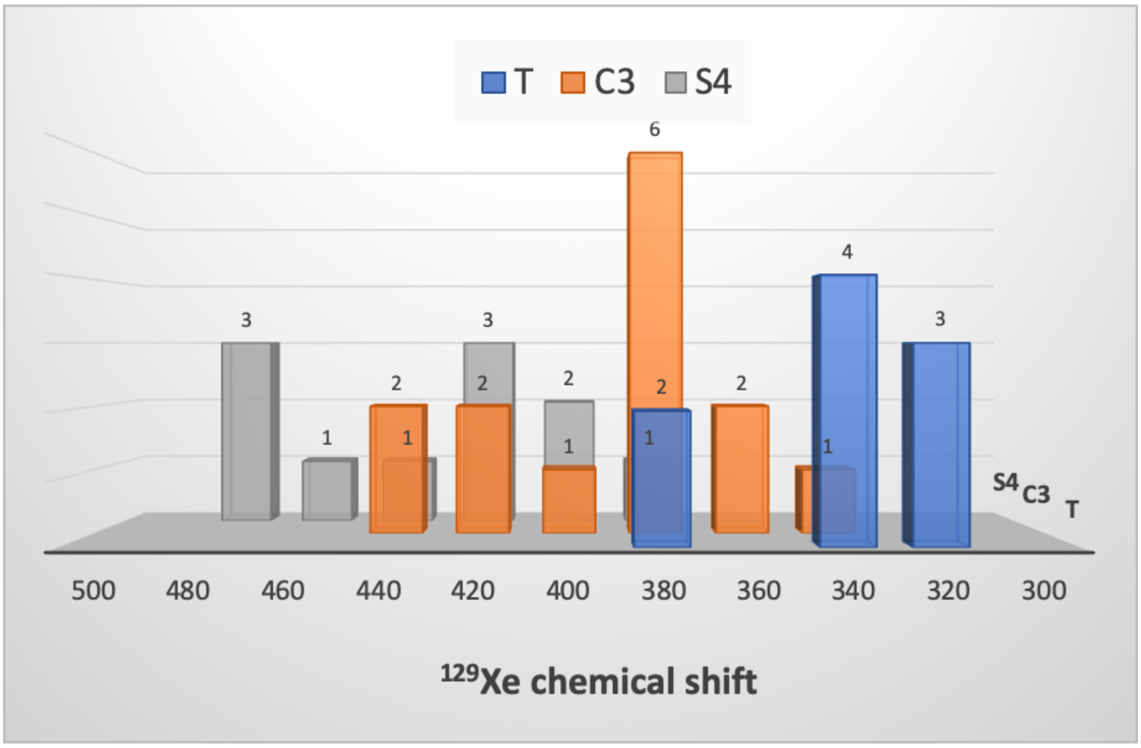

Supplementary Figure 23. Conformer-based distributions of ^{129}Xe chemical shift in different diastereomers of Fe-MOP.

Supplementary Table 3 Computed NMR chemical shifts of the encapsulated ^{129}Xe atom at its equilibrium positions after XTB optimization of different conformations of T, C_3, and S_4 diastereomers of Fe-MOP. In addition, free energy differences (ΔG in kJ/mol) w.r.t. lowest free energy conformation of T diastereomer and the cavity volumes (in Å^3) of different conformers are listed.

	$\sigma(^{129}$**Xe**$)^a$	δ $(^{129}$**Xe**$)^b$	$\Delta_{CS}{}^c$	ΔG	**Volume**^{d}
T-c1	5440.4	**314.1**	0.0	**0.0**	59.5
T-c2	5387.0	367.5	53.4	9.0	82.1
T-c3	5420.3	334.2	20.1	37.6	74.9
T-c4	5424.5	330.0	15.9	48.3	85.4
T-c5	5447.4	307.1	-7.0	50.6	66.3
T-c6	5383.1	371.5	57.3	67.5	48.4
T-c7	5422.3	332.2	18.1	70.1	60.7
T-c8	5446.9	307.6	-6.5	93.2	79.4
T-c9	5428.1	326.5	12.3	104.4	71.8
C_3-*c1*	5396.5	**358.1**	44.0	**0.6**	45.6
C_3-*c2*	5391.3	363.2	49.1	4.9	71.9
C_3-*c3*	5330.3	424.2	110.1	14.4	81.8
C_3-*c4*	5401.9	352.6	38.5	24.9	69.2
C_3-*c5*	5322.9	431.6	117.5	33.8	62.1
C_3-*c6*	5392.1	362.4	48.3	37.8	64.3
C_3-*c7*	5354.5	400.0	85.9	38.6	53.5
C_3-*c8*	5348.0	406.6	92.5	41.9	74.4
C_3-*c9*	5415.4	339.1	25.0	43.6	63.2
C_3-*c10*	5391.9	362.7	48.6	55.9	70.6
C_3-*c11*	5370.0	384.6	70.5	94.5	64.6
C_3-*c12*	5378.2	376.3	62.2	98.9	61.0
C_3-*c13*	5378.0	376.5	62.4	116.7	53.8
C_3-*c14*	5394.4	360.1	46.0	122.1	48.9
S_4-*c1*	5372.1	**382.4**	68.3	**43.0**	61.4
S_4-*c2*	5279.3	475.3	161.2	49.8	75.5
S_4-*c3*	5338.4	416.1	102.0	51.6	47.0
S_4-*c4*	5368.2	386.4	72.3	51.7	62.7
S_4-*c5*	5288.8	465.7	151.6	53.0	68.1
S_4-*c6*	5323.4	431.1	117.0	58.6	126.3
S_4-*c7*	5286.2	468.3	154.2	59.1	52.0
S_4-*c8*	5352.2	402.3	88.2	65.7	48.2
S_4-*c9*	5390.4	364.1	50.0	76.4	71.8
S_4-*c10*	5314.1	440.4	126.3	91.7	68.9
S_4-*c11*	5338.1	416.5	102.4	115.3	68.5

[a] NMR nuclear shielding constants were computed with Amsterdam Density Functional (ADF) code [19–21] at scalar-relativistic zeroth-order (SR-ZORA) level [22] using density functional theory (DFT) with BHandHLYP functional [23, 24] and QZ4P-J(Xe)/TZP(Fe)/DZ(other) basis sets. [25] Spherical Gaussian model was used for nuclear charge distribution. [26] Numerical quality was set to VeryGood for the Xe atom, Normal for the atoms of the complex and Basic for the solvent molecules.
[b]Chemical shifts computed are referenced to the nuclear shielding value of 5754.5 ppm for Xe atom computed at the same level of theory corresponding to an internal low-density gas reference.
[c]Chemical shift differences with respect to T diastereomer
[d]Cavity volume was computed with Caver analyst code [27] using probe radius of 1.2 Å with 100000 samples. The cavity volume of the X-ray structure [1] is 132 Å^3 (in the cited article the value is slightly larger, 141 Å^3, due to the different analysis program).

Supplementary Table 4 Computed ^{129}Xe NMR chemical shifts for the lowest free energy $(\min - G)$ conformation as well as averages, minima, maxima, and standard deviations of the shift distributions (Supplementary Table 3) for encapsulated Xe inside different conformations of T, C_3, and S_4 diastereomers of Fe-MOP.

	T	C_3	S_4	C_3-T	S_4-T	S_4-C_3
$\delta^{\min-G}$	314.1	358.1	382.4	44	68	24
δ^{ave}	332.3	378.4	422.6	46	90	44
$\delta^{\min}$	307.1	339.1	364.1	32	57	25
$\delta^{\max}$	371.5	431.6	475.3	60	104	44
δ^{stdev}	23.4	27.6	37.3			

The data of Supplementary Table 4 clearly shows that the ^{129}Xe chemical shifts distributions of different diastereomers are overlapping. However, conformers of T diastereomer display lower chemical shift range than those of C_3 and S_4. A similar trend can also be seen in chemical shift averages of each diastereomer: $\delta^{\text{ave}}(T) < \delta^{\text{ave}}(C_3) < \delta^{\text{ave}}(S_4)$ and for the lowest free energy (min-G) conformations: $\delta^{\min-G}(T) < \delta^{\min-G}(C_3) < \delta^{\min-G}(S_4)$. As the latter are the most plausible structures making their Xe chemical shifts the most representative ones, we chose them for more detailed scrutiny.

13.3 Thermal averages of ^{129}Xe chemical shifts in the lowest G conformations

It is obvious from the data in Supplementary Tables 3 and 4 that ^{129}Xe chemical shifts in preliminary GFN2-xTB structures are in much higher range than the experimental ones around 200 ppm. This overestimation is probable due to previously found underestimation of repulsive interactions [28]. Consequences are too compact cavity geometries as well as too strong Xe atom attractive interaction with cage atoms. Both of these make Xe atom to be at too close contact with the cavity walls, which lead to overestimation of ^{129}Xe chemical shifts. However, the error is expected to be similar in different systems and, hence, relative chemical shift differences between conformations and diastereomers are qualitatively correct, while probably slightly overestimated due to above reasons.

In order to improve estimations of the ^{129}Xe shifts inside the lowest free energy $(\min - G)$ conformers of each diastereomer, (*T-c1*, *C3-c1*, and *S4-c1*), we first removed all 36 explicit water molecules from each cluster and then carried out geometry optimization at density functional theory (DFT) level of theory. At the obtained equilibrium geometries, NMR shieldings were computed for ^{129}Xe atom only using efficient hybrid DFT implementation of Turbomole code [29]. ^{129}Xe chemical shifts at the equilibrium geometries ($\delta_{\text{Xe}}^{r_e}$) are displayed in Supplementary Table 5 (see technical details in footnotes).

The modeling above lacks effects due to thermal motions of atoms. As the current supramolecular Xe@$Fe_4L_6^{4-}$ complex including four NH_4^+ counter ions is already quite large, full treatment of its molecular dynamics (MD) at finite temperature in explicit solvent of hundred of water molecules is very challenging. There exists no accurate enough classical force-fields for the current complicated supramolecular structure with several flexible ligands and charged SO_3 groups. Instead, reliable enough dynamics and structures for sensitive Xe chemical shift averaging preferably requires DFT or at least, much faster, semi-empirical, *e.g.* GFN2-xTB, MD simulations. However, short trials with the latter method for finite droplet including the complex and 500 water molecules indicated that long enough trajectories for reasonable statistics are not feasible with currently available computing resources.

Therefore, we focus on treatment of the most important effect, *i.e.* Xe thermal motion inside the rigid DFT optimized equilibrium structures. For that, we used canonical Monte Carlo (MC-NVT) simulations approach introduced in several previous Xe NMR studies of cavity materials [4, 17, 18]. Numerical potential energy (PES) and Xe chemical shift hypersurfaces for *in house* MC-NVT code were generated by moving Xe atom inside the

cage with 0.2 Å displacements in each Cartesian directions around the center of mass of the structure. In each Xe position a fast GFN2-xTB energy calculation was carried out and only points with energy less than 3000 K above the minimum energy were chosen for X2C/DFT/BHandHLYP-D4 level PES and ^{29}Xe chemical shift calculations (see footnotes in Supplementary Table 5). All in all, this resulted in 1374, 772, and 571 calculations for *T-c1*, *C3-c1*, and *S4-c1* systems, respectively. The results at $T = 300$ K ($\delta_{\mathrm{Xe}}^{\mathrm{AVE(300\,K)}}$) are shown in Table 5.

Supplementary Table 5 Computed ^{129}Xe chemical shifts for the lowest free energy (G) conformations of T, C_3, and S_4 diastereomers of Fe-MOP.[a]

	T-c1	***C_3-c1***	***S_4-c1***	***C_3-T***	***S_4-T***	***S_4-C_3***
$\delta_{\mathrm{Xe}}^{r_e\ b}$	149.8	221.3	275.3	72	126	54
$\delta_{\mathrm{Xe}}^{\mathrm{AVE(300\,K)}\ c}$	202.1	254.7	302.5	53	100	48
$\delta_{\mathrm{Xe}}^{\mathrm{EXP}\ d}$	201	212	218	11	17	6

[a] ^{129}Xe shielding tensors were computed for Xe@Fe_4L_6 + 4 NH_4 cluster with Turbomole code [29] at scalar-relativistic X2C level [30]. In hybrid DFT BHandHLYP [23, 24] calculations with D4 dispersion correction [31] x2c-TZVPall-s(Xe)/x2c-SVPall (other) basis sets [32] with finite nucleus model were used. Chemical shifts are referenced to the nuclear shielding value of 5847.6 ppm for Xe atom computed at the same level of theory corresponding to an internal low-density Xe gas reference.
[b] Equilibrium geometry was optimized with Turbomole using DFT/B97-3C composite [33] method with implicit COSMO solvation model of water [34].
[c] MC-NVT averages over Xe motion at $T = 300$ K inside the fixed DFT/B97-3C cages.
[d] Experimental signals at 13, 24 and 30 ppm with respect to Xe in water (188 ppm) [4].

14 ^{1}H NMR spectrum of Fe-MOP with extended scans

^{1}H NMR spectrum Fe-MOP measured with 13312 scans and 5 s recycling delay (Supplementary Figure 24). The major signals in the spectrum arise from T diastereomer. Due to symmetry reasons, each single spectral line corresponding to the T diastereomer should be divided into 4 and 3 lines for C_3 and S_4 diastereomers, respectively. [35] The zoom into baseline level shows minor signals, which (some of them) might arise from the minor diastereomers C_3 and S_4, whose existence was revealed by the ^{129}Xe analysis. However, these signals are partially overlapping with the major signals from T diastereomer, and some signals may be completely hidden under the major peaks. Some of the signals may also arise due to impurities. Therefore, ^{1}H spectrum cannot provide a direct confirmation of the existence of any additional diastereomers. However, it does not exclude it either. We measured ^{1}H-^{13}C HSQC with 512 scans, but it was unable to provide more detailed information about the existence of minor diastereomers.

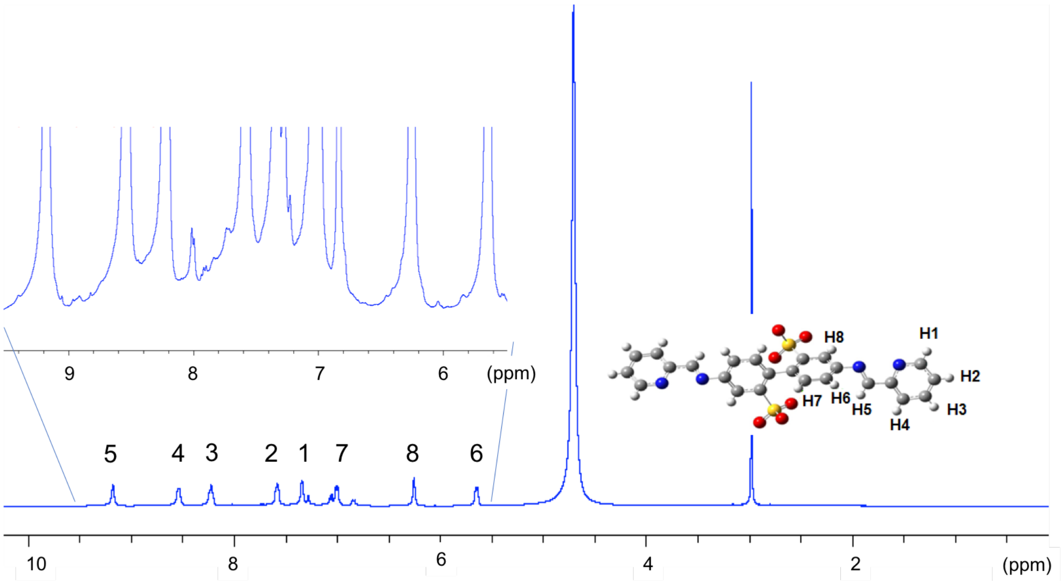

Supplementary Figure 24. ^{1}H NMR spectrum of Fe-MOP sample with Xe. The signals of T diastereomer are labeled in the spectrum. The zoom to the baseline level shows minor peaks that might arise from the minor diastereomers C_3 and S_4, whose existence was revealed by the ^{129}Xe NMR analysis.

15 DFT/B97-3c/COSMO geometries

```
337
T-c1, Turbomole/B97-3c/COSMO optimized
Xe    0.0580671    0.0328900   -0.0364796
Fe   -3.2412440    3.2019192   -6.3183019
Fe    3.8449572    5.3671514    4.0663583
Fe    4.9449134   -5.6687307   -2.2578612
Fe   -5.5941056   -3.1808346    4.4504620
N     3.0308565    6.3747739    5.5398806
N     2.1153610    5.7868700    3.2299632
C     3.5422198    6.6088578    6.7534547
H     4.5214372    6.2127740    6.9629585
C     2.8619152    7.3428958    7.7119270
H     3.3254425    7.5035349    8.6734199
C     1.6078399    7.8569070    7.4211017
H     1.0618484    8.4310713    8.1548320
C     1.0651615    7.6156493    6.1703931
H     0.0869763    7.9820724    5.8979424
C     1.7957395    6.8756109    5.2535784
C     1.3342950    6.5319600    3.9452381
H     0.3697583    6.8650350    3.5842557
C     1.6380610    5.3798165    1.9536886
C     2.3158238    5.7693316    0.8083421
H     3.1922330    6.3885220    0.8635252
C     1.8450483    5.3961603   -0.4346355
C     0.6487133    4.6853367   -0.5665740
C    -0.0089043    4.2986580    0.5942337
H    -0.9412538    3.7604934    0.5133000
C     0.4759087    4.6281158    1.8462441
H    -0.0689546    4.3229505    2.7225817
N    -2.3887586    3.2216978   -8.0874309
N    -1.4250199    3.7100637   -5.7768268
C    -2.9391213    2.9107492   -9.2660886
H    -3.9767059    2.6227723   -9.2674891
C    -2.2238853    2.9658377  -10.4519184
H    -2.7211028    2.7077582  -11.3745620
C    -0.8923052    3.3502339  -10.4334249
H    -0.3182016    3.3986247  -11.3467649
C    -0.3094750    3.6680090   -9.2182254
H     0.7275222    3.9592607   -9.1472825
C    -1.0780059    3.5958627   -8.0668218
C    -0.5874434    3.8656926   -6.7516117
H     0.4383102    4.1689276   -6.5842442
C    -0.9405486    3.9123976   -4.4556854
C    -1.4736297    4.9324218   -3.6832232
H    -2.2313175    5.5869553   -4.0739752
C    -1.0031147    5.1490841   -2.4037200
C     0.0544209    4.3931261   -1.8896007
C     0.5706975    3.3740461   -2.6802620
H     1.3997642    2.7931804   -2.3053865
C     0.0814537    3.1212920   -3.9485555
H     0.5138899    2.3344216   -4.5425786
S     2.9290226    5.7675783   -1.8498098
S    -1.9197310    6.3710440   -1.4119192
O     3.5571787    4.4226656   -2.1297706
O     3.9315799    6.7561897   -1.3389642
O     2.0878577    6.2801822   -2.9675325
O    -0.9380481    7.1857612   -0.6421673
O    -2.7260019    7.1618678   -2.3959952
O    -2.7917743    5.4934588   -0.5473033
N     6.8734066   -5.3920869   -2.5189574
N     4.8821335   -3.9562225   -3.2106547
C     7.8780386   -6.1664967   -2.0952836
H     7.6197718   -7.0652707   -1.5614655
C     9.2058085   -5.8509297   -2.3370491
H     9.9751023   -6.5154495   -1.9741973
C     9.5209262   -4.6966203   -3.0364741
H    10.5494001   -4.4330209   -3.2335526
C     8.4895583   -3.8839336   -3.4759779
H     8.6811407   -2.9676564   -4.0134666
C     7.1817023   -4.2548732   -3.2047509
C     6.0331455   -3.4909050   -3.5784988
H     6.1277693   -2.5658962   -4.1330216
C     3.7293450   -3.1886487   -3.5288578
C     2.7579770   -3.7341051   -4.3542442
H     2.8731043   -4.7169849   -4.7730181
C     1.6374506   -2.9997363   -4.6826667
C     1.4950272   -1.6777017   -4.2519704
C     2.4770290   -1.1525133   -3.4215046
H     2.3864909   -0.1287500   -3.0904574
C     3.5833287   -1.8943672   -3.0473499
H     4.3354679   -1.4551648   -2.4133193
N    -4.9660054    2.6061000   -7.0415498
N    -2.9588700    1.2622609   -6.2214428
C    -6.0050187    3.3626661   -7.4115847
H    -5.9003796    4.4297784   -7.3124355
C    -7.1725402    2.8150249   -7.9191570
H    -7.9777431    3.4744392   -8.2053637
C    -7.2853571    1.4397520   -8.0509476
H    -8.1871143    0.9943133   -8.4440434
C    -6.2192532    0.6456608   -7.6636767
H    -6.2618890   -0.4307960   -7.7311487
C    -5.0773447    1.2529380   -7.1649081
C    -3.9261254    0.5456712   -6.6980581
H    -3.8794680   -0.5355337   -6.7265462
C    -1.8353010    0.5754851   -5.6854491
C    -0.5882957    0.7403680   -6.2694397
H    -0.4571685    1.3655911   -7.1338692
C     0.5052277    0.0673750   -5.7633226
C     0.3655841   -0.8377368   -4.7075658
C    -0.8902870   -0.9810938   -4.1328505
H    -1.0184599   -1.6871985   -3.3262033
C    -1.9855526   -0.2793728   -4.6020969
H    -2.9500005   -0.4247293   -4.1465246
S     0.3228344   -3.8926947   -5.5738782
S     2.1276025    0.5248183   -6.4525258
O     0.9648776   -5.1461074   -6.0833838
O    -0.2098268   -3.0070305   -6.6467334
O    -0.6686301   -4.1757516   -4.4708710
O     2.9135864   -0.7204511   -6.6788581
O     2.6944000    1.4122553   -5.3698246
O     1.8289654    1.2922584   -7.7034981
N    -3.7328882    5.0997874   -6.4155234
N    -4.0604222    3.3973249   -4.5431501
C    -3.4936538    5.9575438   -7.4134999
H    -2.9810861    5.5771494   -8.2808506
C    -3.8954579    7.2827741   -7.3597807
H    -3.6782623    7.9290729   -8.1965673
C    -4.5659456    7.7521205   -6.2410209
H    -4.8864379    8.7815340   -6.1801291
C    -4.8141383    6.8750062   -5.1988426
H    -5.3198734    7.1933993   -4.2999523
C    -4.3880381    5.5607506   -5.3125736
C    -4.5534630    4.5690970   -4.2968353
H    -5.0555310    4.7962124   -3.3648449
C    -4.1658779    2.4138774   -3.5218606
C    -4.8892140    1.2559714   -3.7652329
H    -5.3837337    1.0974153   -4.7059811
C    -5.0131332    0.2984196   -2.7797820
C    -4.4774783    0.5057719   -1.5057530
C    -3.7517306    1.6693053   -1.2848461
H    -3.3414757    1.8490856   -0.3020789
C    -3.5809805    2.6173063   -2.2787123
H    -3.0234183    3.5164616   -2.0752140
N    -6.1062351   -5.0731372    4.3348716
N    -5.6305508   -3.3694070    2.4979644
C    -6.2994811   -5.9327559    5.3412896
H    -6.1732394   -5.5579131    6.3428418
C    -6.6617282   -7.2527111    5.1248406
H    -6.8076174   -7.9009250    5.9755178
C    -6.8300157   -7.7149315    3.8288030
H    -7.1112466   -8.7402783    3.6398776
C    -6.6256527   -6.8364121    2.7783189
H    -6.7281417   -7.1500488    1.7505917
C    -6.2660687   -5.5274011    3.0592741
C    -5.9946191   -4.5346793    2.0671123
H    -6.0779204   -4.7559380    1.0106015
C    -5.3015110   -2.3808756    1.5305539
C    -6.0561811   -1.2195155    1.4579351
H    -6.9012662   -1.0636315    2.1033734
C    -5.7453903   -0.2510702    0.5256136
C    -4.7225447   -0.4529494   -0.4051458
C    -3.9770136   -1.6209495   -0.3117023
H    -3.1922534   -1.7974472   -1.0322290
C    -4.2466473   -2.5782768    0.6501728
H    -3.6606474   -3.4808768    0.6896777
S    -5.7777889   -1.2785458   -3.2768314
S    -6.6353212    1.3308665    0.6737963
O    -6.4398959   -1.0038957   -4.5918881
O    -6.7171659   -1.7106047   -2.2044724
O    -4.5751561   -2.1788802   -3.4291924
O    -7.0316590    1.7802983   -0.6905422
O    -5.6033162    2.2157530    1.3293403
O    -7.7934861    1.0549544    1.5828446
N     4.5998289    6.9776806    3.2384924
N     4.6551178    4.5412035    2.4811357
C     4.4936233    8.2425118    3.6597095
H     3.9347036    8.4183375    4.5631223
C     5.0861293    9.2972242    2.9833860
H     4.9701698   10.2977865    3.3713603
C     5.8137756    9.0510754    1.8296138
H     6.2823625    9.8596920    1.2886311
C     5.9247579    7.7461417    1.3798990
H     6.4674174    7.5025209    0.4791551
C     5.3101300    6.7341227    2.1007904
C     5.3172234    5.3557770    1.7225974
H     5.8368188    5.0193188    0.8343915
C     4.5964989    3.1789235    2.0770858
C     5.1594426    2.2015063    2.8832036
H     5.6458337    2.4570849    3.8070949
C     5.1503434    0.8821145    2.4768341
C     4.6278451    0.5155662    1.2339332
C     4.0551514    1.5090306    0.4500083
H     3.6612274    1.2456075   -0.5198468
C     4.0282457    2.8295750    0.8592004
H     3.5965282    3.5796577    0.2185258
N     5.2198951   -7.3297194   -1.2466855
N     5.0996955   -4.9032783   -0.4556782
C     5.2264734   -8.5836095   -1.7117772
H     5.0652962   -8.7214857   -2.7673537
C     5.4462110   -9.6753937   -0.8866628
H     5.4437356  -10.6648293   -1.3179900
C     5.6653316   -9.4800714    0.4678887
H     5.8378930  -10.3180496    1.1266500
C     5.6536576   -8.1868523    0.9625213
H     5.8045781   -7.9812770    2.0114091
C     5.4302008   -7.1366424    0.0859680
C     5.3582281   -5.7638285    0.4755672
H     5.4970699   -5.4724560    1.5077119
C     4.9492222   -3.5416037   -0.0737711
C     5.8398529   -2.5910067   -0.5491170
H     6.6607072   -2.8682168   -1.1859174
C     5.7003796   -1.2674402   -0.1812247
C     4.7152881   -0.8729758    0.7287530
C     3.8259781   -1.8380573    1.1844433
H     3.0552543   -1.5512290    1.8851961
C     3.9259259   -3.1587912    0.7841890
H     3.2127856   -3.8829125    1.1381933
S     5.7633005   -0.3322487    3.6853067
S     6.7648886   -0.0715955   -1.0520366
O     6.5124250    0.4610131    4.7062892
O     6.5842682   -1.3530718    2.9718405
O     4.4812412   -0.9169378    4.2500096
O     7.8314868   -0.8907731   -1.7099465
O     7.2800976    0.9110352   -0.0585045
O     5.8072623    0.5220118   -2.0562399
N     5.4891149    5.0067818    5.0777176
N     3.3027520    3.6816984    4.9400372
C     6.6244691    5.7140400    5.0763171
H     6.6575336    6.5971943    4.4612965
C     7.7214995    5.3513594    5.8413691
H     8.6101957    5.9627900    5.8029992
C     7.6585110    4.2195112    6.6390311
H     8.5027995    3.9204676    7.2422315
C     6.4905196    3.4763312    6.6451940
H     6.3934666    2.5806009    7.2398602
C     5.4269066    3.8912984    5.8581610
C     4.1843265    3.1948236    5.7556698
H     3.9995002    2.2955703    6.3293492
C     2.0953831    2.9495196    4.7809849
C     0.8793995    3.5678820    5.0321887
H     0.8330491    4.5890175    5.3604723
C    -0.2980084    2.8607543    4.9028533
C    -0.2891157    1.5002954    4.5845982
C     0.9388432    0.8964119    4.3409112
H     0.9690117   -0.1593025    4.1164847
C     2.1240398    1.6061855    4.4229163
H     3.0589180    1.1066367    4.2250017
N    -7.4526406   -2.5519841    4.4134422
N    -5.2642700   -1.2561371    4.4753744
C    -8.5655043   -3.2889829    4.3317041
H    -8.4479949   -4.3586040    4.2870803
C    -9.8283806   -2.7183065    4.3204858
H   -10.6924114   -3.3620404    4.2559842
C    -9.9596401   -1.3402799    4.3924521
H   -10.9350726   -0.8770357    4.3845569
C    -8.8138516   -0.5663393    4.4696226
H    -8.8608781    0.5111644    4.5118938
C    -7.5792683   -1.1958854    4.4776248
C    -6.3223941   -0.5133556    4.5158800
H    -6.2649661    0.5669322    4.5576987
C    -3.9960444   -0.6098872    4.4716999
C    -3.1858990   -0.7069990    5.5928251
H    -3.5092644   -1.2428617    6.4679361
C    -1.9585757   -0.0762428    5.6132480
C    -1.5399371    0.7099360    4.5361512
C    -2.3634034    0.7840944    3.4203222
H    -2.0612770    1.4021663    2.5886271
C    -3.5808083    0.1277348    3.3732356
H    -4.2114596    0.2181523    2.5055692
S    -1.8389809    3.8228953    5.0434950
S    -0.8954216   -0.3967922    7.0556680
O    -2.2704734    3.9354093    3.6007569
O    -1.4310928    5.1482052    5.6129097
O    -2.7981307    3.0676710    5.8958414
O    -0.3817428    0.9229367    7.5363805
O     0.1742754   -1.2960698    6.5105673
O    -1.7849540   -1.0838504    8.0523147
N    -5.5355396   -3.2055638    6.4144111
N    -3.7164805   -3.6953440    4.6943279
C    -6.5194867   -2.9007493    7.2674633
H    -7.4702915   -2.6206272    6.8467489
C    -6.3470257   -2.9528681    8.6416611
H    -7.1773712   -2.6983744    9.2826377
C    -5.1201581   -3.3289246    9.1656820
H    -4.9658850   -3.3744080   10.2335217
C    -4.0925795   -3.6423677    8.2922216
H    -3.1143120   -3.9265282    8.6487594
C    -4.3272727   -3.5713824    6.9279621
C    -3.3433730   -3.8368637    5.9258019
H    -2.3363267   -4.1337347    6.1901353
C    -2.7418820   -3.9036152    3.6814827
C    -2.9308287   -4.9227858    2.7603750
H    -3.7901524   -5.5663119    2.8139988
C    -1.9934403   -5.1448913    1.7725927
C    -0.8113904   -4.3996089    1.7246424
C    -0.6441931   -3.3802745    2.6545347
H     0.2681307   -2.8045820    2.6401294
C    -1.5986971   -3.1170029    3.6222476
H    -1.4461338   -2.3202960    4.3333766
N     4.7331138   -6.6234189   -3.9557039
N     3.0033056   -5.9665492   -2.1994724
C     5.6764903   -6.8959438   -4.8637040
H     6.6775169   -6.5591433   -4.6536013
C     5.3995252   -7.5967776   -6.0267132
H     6.2011153   -7.7916075   -6.7228375
C     4.1075333   -8.0340394   -6.2738683
H     3.8723940   -8.5819015   -7.1742155
C     3.1206971   -7.7501007   -5.3450562
H     2.0962374   -8.0537493   -5.4974089
C     3.4599324   -7.0457300   -4.2004990
C     2.5297315   -6.6573533   -3.1867072
H     1.4807194   -6.9162044   -3.2556307
C     2.0863588   -5.5134610   -1.2115861
C     2.2249346   -5.9508576    0.0970927
H     3.0048069   -6.6359154    0.3744846
C     1.3422221   -5.5224807    1.0666134
C     0.2477980   -4.7165156    0.7385527
C     0.1364889   -4.2793305   -0.5749043
H    -0.7045333   -3.6594584   -0.8480316
C     1.0466379   -4.6571165   -1.5470694
H     0.9236926   -4.3129313   -2.5601597
S    -2.4643862   -6.3300016    0.4698408
S     1.7450058   -5.9638385    2.7848718
O    -3.6259422   -7.0948562    1.0256569
O    -1.2837189   -7.1762503    0.1415412
O    -2.8823408   -5.4093966   -0.6521936
O     0.5585479   -6.6390355    3.3783235
O     2.0347394   -4.6225508    3.4263301
O     2.9737711   -6.8162065    2.7015080
N    -4.2309782    4.6359052    1.7431306
H    -4.7775334    3.8108697    1.4562469
H    -3.6850656    5.0068568    0.9543252
H    -3.5979081    4.3847604    2.5197944
H    -4.8694502    5.3559302    2.0605209
N    -3.2148258   -4.6448640   -3.3452637
H    -3.8014193   -3.7992720   -3.2920337
H    -2.3403228   -4.4382565   -3.8487454
H    -3.7216524   -5.3569875   -3.8584157
H    -3.0107376   -5.0030433   -2.4009392
N     4.7648284    2.5028966   -3.7961574
H     4.2884897    3.2659529   -3.2924265
H     5.2038224    1.8566432   -3.1261421
H     5.4899842    2.9036086   -4.3800995
H     4.0957900    2.0026967   -4.3994477
N     4.5228582   -3.6694341    4.1469002
H     5.2519378   -3.8775832    3.4760369
H     3.6169024   -4.0921861    3.8540020
H     4.4512160   -2.6312357    4.2177978
H     4.8014386   -4.0490732    5.0441155
```

```
337
C3-c1, Turbomole/B97-3c/COSMO optimized
Xe   -0.0978493   -0.1907662    0.0340382
Fe    5.2817932    3.4784879    4.0506759
Fe    1.3691106   -7.6397004    0.0354499
Fe   -1.2218919    2.7484604   -7.0159111
Fe   -6.6607268    1.5185081    3.8560935
N     6.3586745    4.8055088    3.1394085
N     5.9070085    2.3863883    2.5583325
C     6.4927762    6.0964279    3.4637758
H     5.9069424    6.4550295    4.2941452
C     7.3484397    6.9403239    2.7752898
H     7.4216094    7.9736105    3.0790064
C     8.0937846    6.4467212    1.7142542
H     8.7682938    7.0893529    1.1681485
C     7.9496421    5.1163192    1.3592427
H     8.4902097    4.6912914    0.5273330
C     7.0769436    4.3191061    2.0835704
C     6.7855032    2.9480675    1.7909265
H     7.2384699    2.4341009    0.9536585
C     5.4238187    1.0849420    2.2773781
C     5.5276858    0.1050833    3.2531532
H     6.1172122    0.2687861    4.1343218
C     4.8627544   -1.0915436    3.1079811
C     4.1661279   -1.3828870    1.9332166
C     4.1391720   -0.4202756    0.9324468
H     3.6202578   -0.6421981    0.0115656
C     4.7494688    0.8125076    1.0939430
H     4.6821049    1.5502530    0.3123883
N     0.7121675   -8.8440727    1.4458148
N     1.7394813   -6.5115145    1.5995091
C     0.1512551  -10.0496509    1.3019546
H     0.0783503  -10.4463224    0.3035973
C    -0.3095048  -10.7847790    2.3828941
H    -0.7493130  -11.7544590    2.2057773
C    -0.1933380  -10.2688170    3.6639899
H    -0.5466988  -10.8257373    4.5190204
C     0.3868638   -9.0221187    3.8267725
H     0.5005316   -8.5749182    4.8031176
C     0.8294132   -8.3364883    2.7056484
C     1.4386487   -7.0452549    2.7410167
H     1.6350360   -6.5415363    3.6789235
C     2.3182610   -5.2129441    1.6215015
C     3.5938930   -5.0185910    1.1107985
H     4.1641204   -5.8342657    0.7068490
C     4.1605967   -3.7613587    1.1356804
C     3.5044406   -2.6908638    1.7457483
C     2.2254481   -2.9016076    2.2416411
H     1.6990351   -2.0810248    2.7077389
C     1.6230092   -4.1453282    2.1721221
H     0.6257671   -4.2715096    2.5566583
S     4.8072523   -2.1502986    4.5910908
S     5.7123139   -3.5337222    0.2115432
O     4.9041842   -3.5765363    4.1755984
O     5.9442774   -1.6842730    5.4477684
O     3.4721593   -1.7870946    5.1988005
O     6.6722840   -2.7881525    1.0735083
O     5.2587192   -2.7516789   -0.9990817
O     6.1634847   -4.9133218   -0.1543892
N    -0.2124391    1.7929031   -8.3705096
N     0.4959402    2.6602973   -6.0736798
C    -0.6670754    1.2797407   -9.5182907
H    -1.7267341    1.3415118   -9.7010582
C     0.1816666    0.7020788  -10.4487793
H    -0.2356256    0.3037583  -11.3610579
C     1.5443544    0.6494643  -10.1962129
H     2.2211515    0.2085158  -10.9128492
C     2.0220345    1.1626437   -9.0025474
H     3.0702140    1.1225065   -8.7490059
C     1.1239905    1.7226841   -8.1068817
C     1.4661214    2.2013802   -6.8054216
H     2.4797945    2.1271015   -6.4331006
C     0.7922010    2.9020153   -4.7027267
C     0.5628663    4.1409245   -4.1164498
H     0.1526099    4.9529006   -4.6919838
C     0.9467564    4.3625618   -2.8043763
C     1.5768522    3.3625038   -2.0579689
C     1.7508696    2.1182821   -2.6497886
H     2.2370052    1.3350314   -2.0894098
C     1.3739307    1.8826725   -3.9553875
H     1.5523920    0.9148323   -4.3926483
N     4.6738841    4.6403009    5.5212324
N     3.6060682    4.1825006    3.2194743
C     5.2902004    4.8937986    6.6806885
H     6.3064702    4.5521780    6.7845421
C     4.6682792    5.5767889    7.7137252
H     5.2158802    5.7570553    8.6262849
C     3.3606189    6.0110560    7.5586558
H     2.8533275    6.5319626    8.3572084
C     2.7199567    5.7730693    6.3552544
H     1.7032050    6.0911512    6.1809660
C     3.4013669    5.0949579    5.3544652
C     2.8737498    4.8592292    4.0510845
H     1.9189143    5.2819627    3.7683603
C     3.0969008    3.9921964    1.9064003
C     3.9494738    4.0411906    0.8115987
H     4.9889602    4.2739222    0.9284794
C     3.4731287    3.7998422   -0.4631811
C     2.1126645    3.5890611   -0.6957016
C     1.2666664    3.5679258    0.4086301
H     0.2137608    3.3869765    0.2528674
C     1.7410817    3.7478673    1.6936903
H     1.0517288    3.6690155    2.5190945
S     0.6452035    6.0108483   -2.0891085
S     4.7282427    3.6121397   -1.7744957
O     0.1339400    6.8475905   -3.2222998
O     1.9420443    6.4883565   -1.5288083
O    -0.4052984    5.7887450   -1.0318098
O     5.9906003    4.1734986   -1.1951758
O     4.2514980    4.3202520   -2.9936602
O     4.8027062    2.1136914   -1.9450353
N    -7.8281042    0.0279948    4.3283239
N    -5.3805877    0.4795349    4.9767470
C    -9.0717097   -0.2078014    3.8929712
H    -9.4943021    0.4969592    3.1979645
C    -9.8043321   -1.3064469    4.3108002
H   -10.8024696   -1.4459431    3.9242898
C    -9.2500238   -2.1993568    5.2158860
H    -9.8076095   -3.0587499    5.5574642
C    -7.9632990   -1.9698249    5.6687843
H    -7.4840813   -2.6378975    6.3691064
C    -7.2747023   -0.8567337    5.2042336
C    -5.9288195   -0.5508819    5.5503468
H    -5.3879630   -1.1773502    6.2470204
C    -3.9855620    0.6518618    5.1256886
C    -3.4192272    1.9183882    5.0424143
H    -4.0348774    2.7931863    4.9922818
C    -2.0479995    2.0724425    4.9790376
C    -1.1955019    0.9707105    5.0858655
C    -1.7783630   -0.2852474    5.1972344
H    -1.1560160   -1.1605334    5.2178508
C    -3.1449828   -0.4593959    5.1986850
H    -3.5350858   -1.4651418    5.1527853
N     6.8169428    2.8516114    5.1044164
N     4.3814438    2.0323527    5.0844363
C     8.0867396    3.2664053    5.0353393
H     8.3217872    4.0329401    4.3176267
C     9.0777786    2.7576727    5.8600624
H    10.0837680    3.1363106    5.7616686
C     8.7620591    1.7810341    6.7913082
H     9.5199169    1.3716982    7.4426183
C     7.4533116    1.3372341    6.8667929
H     7.1549581    0.5712319    7.5665594
C     6.5053956    1.8889195    6.0170461
C     5.1400792    1.4814308    5.9811211
H     4.7883996    0.6893930    6.6268901
C     3.0142123    1.6786601    5.0733592
C     2.3424147    1.4854917    6.2780895
H     2.8499815    1.6085505    7.2222199
C     1.0048995    1.1567816    6.2794676
C     0.2784988    1.1081143    5.0868692
C     0.9785544    1.2363521    3.8926457
H     0.4386916    1.1648731    2.9605545
C     2.3358140    1.4974127    3.8767517
H     2.8613536    1.5665339    2.9401673
S    -1.4414742    3.7459859    4.5859804
S     0.2574276    0.6989586    7.8708989
O    -2.6076849    4.6616554    4.7833060
O    -1.0760343    3.6196503    3.1154720
O    -0.2608190    4.0425146    5.4408376
O     1.2813310    1.0227019    8.9122564
O     0.0347679   -0.7919443    7.7331089
O    -1.0168974    1.4574689    8.0222049
N     3.1998111   -8.3126798    0.1088387
N     2.1796913   -6.4365602   -1.2846331
C     3.6879601   -9.2618949    0.9143365
H     3.0109910   -9.6997029    1.6285965
C     5.0039931   -9.6882580    0.8343511
H     5.3452800  -10.4607905    1.5064978
C     5.8535729   -9.1210494   -0.1030704
H     6.8815383   -9.4421326   -0.1821670
C     5.3615535   -8.1276895   -0.9328402
H     5.9877001   -7.6407033   -1.6647321
C     4.0362363   -7.7425205   -0.8045739
C     3.4203849   -6.6923911   -1.5551950
H     3.9794199   -6.1202671   -2.2845662
C     1.5881074   -5.3096930   -1.9223337
C     0.5200859   -5.4791018   -2.7907486
H     0.1341821   -6.4602153   -3.0092332
C    -0.0346112   -4.3809197   -3.4217405
C     0.4835392   -3.0977837   -3.2238198
C     1.5511462   -2.9531558   -2.3492816
H     1.9811482   -1.9732327   -2.2075983
C     2.1060763   -4.0399665   -1.7024247
H     2.9494790   -3.8988424   -1.0504441
N    -2.9326048    2.6654899   -8.0017378
N    -1.8937056    1.0337867   -6.3230864
C    -3.4273626    3.5428501   -8.8819493
H    -2.7998569    4.3699310   -9.1656372
C    -4.6881923    3.4026335   -9.4408238
H    -5.0304639    4.1418270  -10.1489584
C    -5.4779839    2.3194202   -9.0903066
H    -6.4639045    2.1931243   -9.5122748
C    -4.9715507    1.3942149   -8.1934154
H    -5.5408264    0.5252701   -7.8977271
C    -3.7023035    1.5906958   -7.6701402
C    -3.0586083    0.6863773   -6.7732730
H    -3.5139932   -0.2594775   -6.5079430
C    -1.2361736    0.0961692   -5.4747410
C    -0.0283936   -0.4565148   -5.8740210
H     0.4388490   -0.1718414   -6.7979433
C     0.5767730   -1.4271624   -5.1015403
C    -0.0487863   -1.9286060   -3.9582025
C    -1.2363171   -1.3317958   -3.5529676
H    -1.7220403   -1.6840345   -2.6556984
C    -1.8252469   -0.3188605   -4.2880606
H    -2.7537021    0.1130466   -3.9550388
S    -1.4910242   -4.6834212   -4.4763736
S     2.2762900   -1.8842165   -5.5685197
O    -1.2575055   -3.9983393   -5.7784344
O    -2.6411452   -4.1023528   -3.6890070
O    -1.5960108   -6.1745636   -4.5761504
O     2.4421757   -3.3558075   -5.4147502
O     3.0878168   -1.0748293   -4.5817385
O     2.4544969   -1.3935303   -6.9714514
N    -8.0378598    2.3352808    2.7170038
N    -6.2179828    0.6188298    2.1774343
C    -8.9720744    3.2233753    3.0690900
H    -9.0534214    3.4645330    4.1162653
C    -9.8173880    3.8056782    2.1375332
H   -10.5575322    4.5156698    2.4739116
C    -9.6936143    3.4720999    0.7978300
H   -10.3322913    3.9271293    0.0558901
C    -8.7327277    2.5485096    0.4252007
H    -8.5834231    2.2642914   -0.6051717
C    -7.9217816    1.9950348    1.4036736
C    -6.9202550    1.0055604    1.1569047
H    -6.7494995    0.6232933    0.1593794
C    -5.3716784   -0.5047669    1.9836755
C    -5.9176508   -1.6626391    1.4444388
H    -6.9764992   -1.7197779    1.2431215
C    -5.1146764   -2.7580204    1.1826499
C    -3.7479264   -2.7370330    1.4871954
C    -3.2310871   -1.5785454    2.0553222
H    -2.1848608   -1.5495349    2.3165057
C    -4.0212100   -0.4760346    2.2944267
H    -3.5743984    0.4072034    2.7004534
N     0.9118833   -8.9087687   -1.3957060
N    -0.5098628   -7.0771190   -0.2757578
C     1.7055171   -9.8064644   -1.9908308
H     2.7312722   -9.8550398   -1.6694368
C     1.2439094  -10.6644496   -2.9763451
H     1.9313383  -11.3698751   -3.4177523
C    -0.0832239  -10.6066270   -3.3717107
H    -0.4625737  -11.2682532   -4.1361803
C    -0.9139466   -9.6797435   -2.7663227
H    -1.9543925   -9.5891906   -3.0404877
C    -0.3908185   -8.8470436   -1.7876415
C    -1.1385635   -7.8291065   -1.1273274
H    -2.1857918   -7.6800015   -1.3564104
C    -1.2347391   -5.9615835    0.2185105
C    -1.3206658   -5.6784345    1.5744207
H    -0.8423375   -6.3062476    2.3030744
C    -2.0891027   -4.6148326    2.0216553
C    -2.8327218   -3.8390928    1.1248655
C    -2.6934799   -4.1170102   -0.2316674
H    -3.2300274   -3.5171425   -0.9471142
C    -1.9212147   -5.1585854   -0.6855322
H    -1.8605670   -5.3401774   -1.7427467
S    -5.9562974   -4.1784227    0.4098019
S    -1.9945146   -4.2723530    3.8131279
O    -5.7067928   -4.0266065   -1.0730597
O    -7.4053588   -4.0131905    0.7487679
O    -5.3474157   -5.4166814    0.9765262
O    -3.3573023   -3.8890688    4.2789274
O    -0.9969349   -3.1341268    3.8952914
O    -1.4542723   -5.5228738    4.4341996
N    -7.1230928    2.5801650    5.4447102
N    -5.6864537    3.1803792    3.4374578
C    -7.8207450    2.1830581    6.5143534
H    -8.2009437    1.1753381    6.5109764
C    -8.0701137    3.0219613    7.5887649
H    -8.6433547    2.6470703    8.4230397
C    -7.5902417    4.3225245    7.5699563
H    -7.7768750    4.9927781    8.3958239
C    -6.8653270    4.7464929    6.4693290
H    -6.4706370    5.7498265    6.4060644
C    -6.6449251    3.8588121    5.4265252
C    -5.9104363    4.1711116    4.2417433
H    -5.5506982    5.1710326    4.0383718
C    -5.1342114    3.4288240    2.1588035
C    -5.8189291    4.2503762    1.2734537
H    -6.7016551    4.7844025    1.5911689
C    -5.4047788    4.3446305   -0.0451076
C    -4.3291087    3.5845795   -0.5066645
C    -3.6092048    2.8291106    0.4189820
H    -2.7488928    2.2707909    0.0816411
C    -3.9900302    2.7672416    1.7419898
H    -3.3969298    2.2261809    2.4550952
N    -0.7348091    4.4544220   -7.8748506
N    -2.1508554    4.0072177   -5.7477559
C     0.0769317    4.6662058   -8.9178800
H     0.5986409    3.8198804   -9.3272203
C     0.2486462    5.9194320   -9.4837245
H     0.9165367    6.0235018  -10.3252146
C    -0.4416772    7.0063459   -8.9712694
H    -0.3285695    7.9892779   -9.4037946
C    -1.2789636    6.8037664   -7.8888067
H    -1.8369033    7.6156645   -7.4463433
C    -1.3988116    5.5253395   -7.3606645
C    -2.1807857    5.2223993   -6.2118437
H    -2.7481488    6.0050180   -5.7263663
C    -2.7204166    3.8372315   -4.4601582
C    -3.4806988    2.7248672   -4.1131883
H    -3.7001894    1.9595924   -4.8318876
C    -4.0154415    2.6152154   -2.8403103
C    -3.8414185    3.6353771   -1.9012822
C    -3.0908470    4.7426914   -2.2697030
H    -2.9291337    5.5365340   -1.5606731
C    -2.5193992    4.8418115   -3.5150078
H    -1.8794496    5.6838429   -3.7256270
S    -6.2771409    5.5486123   -1.1011027
S    -4.9371360    1.0981988   -2.4160469
O    -7.5700965    5.8263507   -0.3965484
O    -6.4509697    4.9235600   -2.4439128
O    -5.3517172    6.7336239   -1.1086719
O    -6.2690761    1.5406926   -1.9052889
O    -4.1001733    0.3656086   -1.4232331
O    -5.0475662    0.3364408   -3.7255996
N     1.0005409   -2.8005752    5.9110170
H     1.1058413   -3.6403684    6.4679322
H     1.9252061   -2.5371479    5.5221640
H     0.6775522   -2.0433982    6.5349917
H     0.3022468   -2.9788873    5.1689651
N    -1.8396843    5.6017237    1.3968341
H    -1.5576131    4.8503642    2.0718458
H    -1.1929111    5.6607942    0.5957230
H    -1.8469685    6.4975811    1.8717542
H    -2.7768123    5.4143989    1.0570628
N     5.2471492   -0.6034358   -2.8462776
H     5.3160839   -1.2602747   -2.0556424
H     6.1208178   -0.6315939   -3.3599506
H     5.0944648    0.3604484   -2.5283677
H     4.4868521   -0.8913454   -3.4826502
N    -4.9228059   -2.4935757   -3.3630613
H    -4.0191069   -2.9273884   -3.6030858
H    -5.2157065   -2.8254384   -2.4343705
H    -5.6128178   -2.8093252   -4.0350229
H    -4.8858395   -1.4595177   -3.3984928
```

```
337
S4-c1, Turbomole/B97-3c/COSMO optimized
Xe    0.3465751    0.1628123   -0.0304903
Fe    0.1219745    4.5888324    6.1789777
Fe    5.7305417   -5.1862092    1.6685085
Fe    2.3283116    4.4095283   -6.1199034
Fe   -6.4865103   -3.0348073   -1.1646383
N     0.7105064    6.5679921    6.0392253
N    -0.8864821    5.2140647    4.4608389
C     1.4858781    7.2187467    6.8680824
H     1.8611295    6.6751370    7.7099398
C     1.8137251    8.5493430    6.6741722
H     2.4507301    9.0456078    7.3754030
C     1.3117307    9.1982213    5.5796540
H     1.5490054   10.2258381    5.3971222
C     0.4977764    8.5037533    4.6980688
H     0.1147209    8.9645784    3.8138572
C     0.2217591    7.1980794    4.9661617
C    -0.6162228    6.3757666    4.0833239
H    -0.9642132    6.7915528    3.1545883
C    -1.6557801    4.3042527    3.7007117
C    -2.6129151    3.6023440    4.4036319
H    -2.8302033    3.8722411    5.4065740
C    -3.2130485    2.5072755    3.8551957
C    -2.9423047    2.1455304    2.5417886
C    -2.0481181    2.9058848    1.8243375
H    -1.8337124    2.6568595    0.8073801
C    -1.3766328    3.9752347    2.3989840
H    -0.6317718    4.5002034    1.8380198
N    -6.7087612   -4.8087402   -0.1258119
N    -5.5497227   -2.5410273    0.6848428
C    -7.2745434   -5.9118977   -0.5472133
H    -7.6773932   -5.9053823   -1.5373543
C    -7.3569343   -7.0431415    0.2443241
H    -7.8273761   -7.9236312   -0.1390755
C    -6.8278408   -7.0063189    1.5059258
H    -6.8706734   -7.8655343    2.1425876
C    -6.2290170   -5.8377548    1.9493073
H    -5.7874217   -5.7704431    2.9205733
C    -6.1974589   -4.7678747    1.1047886
C    -5.5621528   -3.5057701    1.4880784
H    -5.1059415   -3.4507693    2.4594476
C    -4.8597958   -1.3628008    1.0828514
C    -5.4837346   -0.1405299    0.9512802
H    -6.4626851   -0.0776999    0.5379885
C    -4.8476694    1.0026272    1.3626858
C    -3.5931446    0.9471589    1.9462246
C    -2.9732576   -0.2766867    2.0425573
H    -2.0090565   -0.3373132    2.5016628
C    -3.5874452   -1.4303624    1.5996693
H    -3.0716923   -2.3666780    1.6664341
S    -4.1070382    1.4679380    4.9967605
S    -5.6616272    2.5595136    1.0723304
O    -5.3483304    1.0211632    4.3847883
O    -4.3305163    2.3224123    6.1672903
O    -3.1854914    0.3762731    5.2895995
O    -5.8268513    3.2478596    2.3410236
O    -4.7868369    3.2948116    0.1549712
O    -6.9348695    2.2014475    0.4454529
N    -1.4803919    4.9733327    7.4461059
N    -0.7347259    2.6704612    6.3937575
C    -1.8577118    6.1375042    7.9165534
H    -1.3152275    6.9958159    7.5830773
C    -2.9035250    6.2648113    8.8105688
H    -3.1755322    7.2338095    9.1721964
C    -3.5684595    5.1373178    9.2140134
H    -4.3823237    5.2005513    9.9063035
C    -3.1773909    3.9101171    8.7061343
H    -3.6821892    3.0067471    8.9746167
C    -2.1345914    3.8752379    7.8277335
C    -1.6608478    2.6211831    7.2356275
H    -2.1268652    1.7018476    7.5298135
C    -0.1873175    1.4710385    5.8897295
C     0.2760666    0.5174734    6.7644952
H     0.1861496    0.6575367    7.8193927
C     0.9112283   -0.5993961    6.2812071
C     1.1167415   -0.7665322    4.9248355
C     0.6038691    0.1791493    4.0656189
H     0.7555469    0.0576621    3.0129831
C    -0.0457815    1.2950367    4.5377041
H    -0.4082404    2.0368048    3.8616969
N     5.5883483   -7.2221720    2.1545859
N     4.0431722   -5.2122765    2.9906734
C     6.3726466   -8.1994479    1.7695839
H     7.2570480   -7.9354497    1.2327034
C     6.0900677   -9.5258153    2.0387802
H     6.7621988  -10.2868857    1.7033738
C     4.9510753   -9.8311571    2.7327718
H     4.6999720  -10.8471944    2.9562726
C     4.1291933   -8.7984200    3.1534565
H     3.2377249   -8.9892740    3.7136108
C     4.4872913   -7.5180115    2.8448329
C     3.6973849   -6.3770809    3.3021625
H     2.8531764   -6.5754716    3.9361690
C     3.2764650   -4.1257450    3.4775375
C     3.9464493   -2.9631678    3.7837858
H     4.9999322   -2.9129920    3.6866081
C     3.2582625   -1.8590113    4.2066987
C     1.8857243   -1.9151991    4.3847646
C     1.2254936   -3.0707207    4.0398570
H     0.1605087   -3.1104878    4.1041525
C     1.9067245   -4.1726405    3.5685778
H     1.3550081   -5.0240518    3.2282219
S     1.3633271   -1.8362435    7.4685962
S     4.1915192   -0.3501267    4.3491080
O     0.3906834   -2.9239889    7.2521180
O     1.1527542   -1.2095256    8.7725367
O     2.7275336   -2.2656768    7.2369084
O     5.5907623   -0.7690442    4.2190407
O     3.9069781    0.2682948    5.6379418
O     3.7550736    0.4672244    3.2234655
N     1.0750400    3.9714044   -7.7310741
N     1.9879203    2.3187110   -5.8552105
C     0.6222828    4.8072494   -8.6327038
H     1.0917041    5.7666369   -8.6857792
C    -0.4228006    4.4833446   -9.4778929
H    -0.7612693    5.1979164  -10.1979291
C    -1.0105463    3.2520951   -9.3605160
H    -1.8315948    2.9762901   -9.9893534
C    -0.5276588    2.3653640   -8.4122667
H    -0.9648417    1.3997395   -8.2690934
C     0.5152639    2.7649610   -7.6281970
C     1.0803942    1.8850337   -6.6061149
H     0.6896541    0.8866811   -6.5380283
C     2.5262182    1.4778284   -4.8513872
C     3.8460847    1.6572620   -4.5152851
H     4.4398265    2.3557595   -5.0478932
C     4.4112943    0.9340302   -3.4956778
C     3.6724998   -0.0100794   -2.8061279
C     2.3578538   -0.2037805   -3.1767149
H     1.7787836   -0.9467630   -2.6688003
C     1.7763382    0.5345336   -4.1824228
H     0.7443686    0.3797888   -4.4109034
N     7.0107344   -4.6920665    3.2177464
N     6.0218560   -3.0874479    1.3446573
C     7.4494677   -5.4930312    4.1570786
H     7.1222193   -6.5099287    4.1204833
C     8.2994289   -5.0570710    5.1566384
H     8.6344048   -5.7451525    5.9035794
C     8.6915189   -3.7455911    5.1645822
H     9.3469486   -3.3752263    5.9253349
C     8.2209573   -2.8980798    4.1744438
H     8.4881431   -1.8628852    4.1508451
C     7.3874316   -3.4121652    3.2255772
C     6.8302611   -2.5783282    2.1582113
H     7.1331129   -1.5507183    2.1099087
C     5.4556509   -2.2710979    0.3329937
C     5.2926361   -2.8244194   -0.9139325
H     5.6396167   -3.8031589   -1.1097815
C     4.6622438   -2.1304557   -1.9099826
C     4.2671442   -0.8197879   -1.7115493
C     4.4493317   -0.2713456   -0.4604254
H     4.1452183    0.7387641   -0.2887421
C     5.0124681   -0.9917293    0.5739836
H     5.0610380   -0.5643206    1.5527973
S     6.1282565    1.2507501   -3.1518252
S     4.2203897   -3.0832514   -3.3436491
O     6.8414820   -0.0001993   -3.3802165
O     6.2155610    1.7139644   -1.7718439
O     6.5005259    2.3013022   -4.1038365
O     5.1427872   -4.2191236   -3.3385421
O     4.3469402   -2.2560489   -4.5402443
O     2.8363241   -3.4681230   -3.1027289
N     4.1300191    4.2045638   -7.0917509
N     3.6883464    4.7172931   -4.5176849
C     4.3173176    3.9378216   -8.3598667
H     3.4508795    3.6971015   -8.9384515
C     5.5744609    3.9667347   -8.9362125
H     5.6852782    3.7428141   -9.9759524
C     6.6519062    4.2855051   -8.1546113
H     7.6379894    4.3171402   -8.5693683
C     6.4504804    4.5641139   -6.8116116
H     7.2661247    4.8068157   -6.1636432
C     5.1770431    4.5171611   -6.3262258
C     4.8797008    4.7642574   -4.9147896
H     5.7075600    4.9307774   -4.2544200
C     3.4274044    4.7363581   -3.1244795
C     2.2226460    5.2214816   -2.6560158
H     1.5174119    5.6464196   -3.3198075
C     1.8896188    5.1064396   -1.3335157
C     2.7854956    4.5657858   -0.4268979
C     3.9792414    4.0799996   -0.9084540
H     4.6658760    3.6054386   -0.2376533
C     4.3028632    4.1494663   -2.2443312
H     5.2078497    3.7022392   -2.5830070
N     1.3608660    3.8968459    7.7235830
N     1.8000774    3.9168893    5.1159386
C     1.1231489    3.9038472    9.0118930
H     0.3442919    4.5497569    9.3591310
C     1.8277944    3.1071191    9.8950996
H     1.5980499    3.1434053   10.9390404
C     2.7976526    2.2742272    9.4041307
H     3.3472367    1.6307198   10.0594009
C     3.0561703    2.2722920    8.0436175
H     3.7838970    1.6244664    7.6042544
C     2.3193310    3.0991294    7.2470042
C     2.5461093    3.1753765    5.7999311
H     3.3540357    2.6050210    5.3806970
C     2.0563443    4.0679487    3.7313520
C     2.2412143    5.3370601    3.2325837
H     2.2378213    6.1796655    3.8870192
C     2.4887200    5.5224448    1.8947330
C     2.5180534    4.4453538    1.0257357
C     2.3641438    3.1815806    1.5540667
H     2.4136833    2.3305716    0.9079743
C     2.1469046    2.9836096    2.8968871
H     2.0745504    1.9964199    3.2820669
S     0.2066391    5.4983329   -0.8954475
S     2.9964625    7.1732792    1.4638559
O     0.1983023    6.2839916    0.3270668
O    -0.4476427    4.1981380   -0.7183791
O    -0.3402329    6.2150724   -2.0492948
O     2.1601926    8.0764444    2.2363850
O     2.9399622    7.3700431    0.0291114
O     4.3963943    7.1911454    1.9419221
N    -8.3366427   -2.3233972   -0.5609240
N    -6.4494899   -1.1040662   -2.0060606
C    -9.2444310   -2.9361964    0.1561496
H    -8.9925256   -3.8994936    0.5436058
C   -10.4837076   -2.3751457    0.4097247
H   -11.1946776   -2.9112684    1.0017665
C   -10.7693725   -1.1418169   -0.1084278
H   -11.7178355   -0.6790208    0.0688751
C    -9.8070941   -0.4943800   -0.8685798
H    -9.9870783    0.4725105   -1.2895597
C    -8.6143746   -1.1236099   -1.0727735
C    -7.5324148   -0.4920190   -1.8257427
H    -7.6831716    0.5166183   -2.1625319
C    -5.3571242   -0.3498500   -2.5095533
C    -4.4877241   -0.8347890   -3.4654250
H    -4.6325525   -1.7918065   -3.9064680
C    -3.4286867   -0.0565586   -3.8788592
C    -3.2326508    1.2068262   -3.3512232
C    -4.0825018    1.6441519   -2.3703645
H    -3.9505860    2.5988866   -1.9326473
C    -5.1440938    0.8886744   -1.9611441
H    -5.8006602    1.2685672   -1.2172762
N     2.3056529    6.4775118   -6.4557138
N     0.5528292    4.9637528   -5.1963222
C     3.2082714    7.2143562   -7.0521878
H     4.0695849    6.7156628   -7.4405810
C     3.0701078    8.5845316   -7.1924283
H     3.8339606    9.1432633   -7.6902136
C     1.9512918    9.1907703   -6.6921931
H     1.8112134   10.2475624   -6.7859059
C     0.9947617    8.4115130   -6.0589297
H     0.1024072    8.8427857   -5.6556644
C     1.2149821    7.0694998   -5.9618906
C     0.2540498    6.1749646   -5.3194756
H    -0.6769041    6.5790728   -4.9685473
C    -0.3984637    4.0582612   -4.6895143
C    -1.6817887    4.0865294   -5.1820264
H    -1.9738064    4.8128491   -5.9081376
C    -2.5963255    3.1724160   -4.7328323
C    -2.2267535    2.1788326   -3.8396614
C    -0.9481785    2.1890034   -3.3364485
H    -0.6675999    1.4651922   -2.6014191
C    -0.0344423    3.1280756   -3.7522426
H     0.9532800    3.1267692   -3.3516768
S    -2.2863304   -0.7507251   -5.0528854
S    -4.2890495    3.5095001   -5.1403189
O    -1.0755715   -1.0598921   -4.2794567
O    -2.0354373    0.2363285   -6.0920818
O    -2.9374337   -1.9654932   -5.5375499
O    -4.2506584    4.4367410   -6.2668643
O    -4.7830530    4.1553898   -3.9131926
O    -4.9802273    2.2698250   -5.4452287
N    -7.2896942   -3.7603718   -2.9503916
N    -4.7683850   -3.7723414   -2.1085863
C    -8.5358888   -3.7322387   -3.3518855
H    -9.2630162   -3.3575574   -2.6647163
C    -8.9174471   -4.1686297   -4.6080800
H    -9.9479925   -4.1273593   -4.8904180
C    -7.9602400   -4.6455245   -5.4607755
H    -8.2201363   -4.9905173   -6.4399090
C    -6.6412500   -4.6783182   -5.0350340
H    -5.8611946   -5.0487631   -5.6667702
C    -6.3558202   -4.2287019   -3.7794974
C    -4.9916462   -4.2368727   -3.2518737
H    -4.2096868   -4.6488522   -3.8641605
C    -3.4301799   -3.8043419   -1.6412805
C    -3.1514098   -4.4364567   -0.4489347
H    -3.9282917   -4.8441487    0.1504196
C    -1.8513503   -4.5989259   -0.0552123
C    -0.8090463   -4.1557159   -0.8573773
C    -1.1171899   -3.4266656   -1.9768908
H    -0.3270063   -3.0492039   -2.5830297
C    -2.4214370   -3.2524641   -2.3819548
H    -2.6284696   -2.7401901   -3.2908543
N     7.2261831   -5.6419231    0.3076914
N     4.6050377   -5.6031148   -0.0482320
C     8.5212412   -5.6149027    0.4972597
H     8.8712285   -5.1817330    1.4101107
C     9.4105535   -6.1300483   -0.4282678
H    10.4610331   -6.0893559   -0.2327282
C     8.9173852   -6.6855327   -1.5779404
H     9.5773048   -7.0955165   -2.3140166
C     7.5462437   -6.7043260   -1.7815599
H     7.1206508   -7.1162762   -2.6724472
C     6.7407508   -6.1781970   -0.8145691
C     5.2881460   -6.0962827   -0.9783849
H     4.8785748   -6.3637989   -1.9305412
C     3.2377492   -5.3051148   -0.2467282
C     2.4556365   -5.9854653   -1.1557917
H     2.8426596   -6.8019096   -1.7221277
C     1.1655743   -5.5850140   -1.3683501
C     0.6067693   -4.5428528   -0.6380026
C     1.4009540   -3.8733034    0.2554607
H     0.9863322   -3.0742224    0.8317761
C     2.7150623   -4.2388919    0.4360197
H     3.3301279   -3.6809886    1.0996954
S    -1.5881234   -5.2886791    1.5602854
S     0.2911958   -6.3845086   -2.6927841
O    -0.5733558   -6.3293782    1.4603682
O    -2.8974548   -5.8092403    1.9659891
O    -1.1689171   -4.1570056    2.3818768
O     1.1787548   -7.4502658   -3.1604442
O    -0.9619252   -6.9000018   -2.1564622
O     0.0767408   -5.3618395   -3.7190311
N     0.9567257   -2.9408410   -5.0003224
H     1.8728920   -2.5674866   -4.8105812
H     0.2360284   -2.2574822   -4.7816219
H     0.8478494   -3.7677513   -4.4233530
H     0.8911173   -3.2021137   -5.9679866
N    -3.1172028    4.8222282   -1.6410351
H    -2.1921024    4.4318934   -1.4929451
H    -3.4878587    4.6177817   -2.5635715
H    -3.0498585    5.8200635   -1.5410532
H    -3.7570110    4.4576079   -0.9449961
N     6.4089700    5.3927038    1.5453747
H     5.6337603    6.0548253    1.6459005
H     6.0895847    4.4484477    1.6713421
H     7.1060699    5.5817109    2.2462296
H     6.8358258    5.4881835    0.6399907
N    -1.5334076   -2.1673601    9.0842560
H    -2.0351890   -2.8902837    9.5699898
H    -0.9439531   -1.6670465    9.7269527
H    -2.1976722   -1.5354096    8.6725173
H    -0.9285700   -2.5685696    8.3666661
```

Supplementary References

[1] Prasenjit Mal, David Schultz, Kodiah Beyeh, Kari Rissanen, and Jonathan R. Nitschke. An Unlockable-Relockable Iron Cage by Subcomponent Self-Assembly. *Angew. Chem. Int. Ed.*, 47(43):8297–8301, 2008.

[2] Shigang Wan, Li-Rong Lin, Lili Zeng, Yiji Lin, and Hui Zhang. Efficient optical resolution of water-soluble self-assembled tetrahedral M_4L_6 cages with 1,1'-bi-2-naphthol. *Chem. Commun.*, 50(97):15301–15304, 2014.

[3] Salvatore Zarra, Maarten M. J. Smulders, Quentin Lefebvre, Jack K. Clegg, and Jonathan R. Nitschke. Guanidinium Binding Modulates Guest Exchange within an [M4l6] Capsule. *Angew. Chem. Int. Ed.*, 51(28):6882–6885, 2012.

[4] Juho Roukala, Jianfeng Zhu, Chandan Giri, Kari Rissanen, Perttu Lantto, and Ville-Veikko Telkki. Encapsulation of Xenon by a Self-Assembled Fe_4L_6 Metallosupramolecular Cage. *J. Am. Chem. Soc.*, 137(7):2464–2467, 2015.

[5] M.H. Levitt. *Spin dynamics: basics of nuclear magnetic resonance*. John Wiley and Sons, Chichester, England, Hoboken, NJ, second edition edition, 2008.

[6] Nir London and Xavier Ambroggio. An accurate binding interaction model in de novo computational protein design of interactions: If you build it, they will bind. *J. Struct. Biol.*, 185(2):136–146, 2014.

[7] Christoph Bannwarth, Sebastian Ehlert, and Stefan Grimme. GFN2-xTB—An Accurate and Broadly Parametrized Self-Consistent Tight-Binding Quantum Chemical Method with Multipole Electrostatics and Density-Dependent Dispersion Contributions. *Journal of Chemical Theory and Computation*, 15(3):1652–1671, March 2019.

[8] xTB standalone code, version 6.4.1 please contact xtb@thch.uni-bonn.de for the program.

[9] Peter A. Kollman, Irina Massova, Carolina Reyes, Bernd Kuhn, Shuanghong Huo, Lillian Chong, Matthew Lee, Taisung Lee, Yong Duan, Wei Wang, Oreola Donini, Piotr Cieplak, Jaysharee Srinivasan, David A. Case, and Thomas E. Cheatham. Calculating Structures and Free Energies of Complex Molecules: Combining Molecular Mechanics and Continuum Models. *Accounts of Chemical Research*, 33(12):889–897, December 2000.

[10] Rocco Meli and Philip C. Biggin. spyrmsd: symmetry-corrected RMSD calculations in python. *J. Cheminformatics*, 12(1):49, 2020.

[11] H. W. Kuhn. The hungarian method for the assignment problem. *Nav. Res. Logist. Q.*, 2(1):83–97, 1955.

[12] William J. Allen and Robert C. Rizzo. Implementation of the hungarian algorithm to account for ligand symmetry and similarity in structure-based design. *J. Chem. Inf. Model.*, 54(2):518–529, 2014.

[13] Thierry Brotin and Jean-Pierre Dutasta. Xe@cryptophane Complexes with C2 Symmetry: Synthesis and Investigations by 129xe NMR of the Consequences of the Size of the Host Cavity for Xenon Encapsulation. *Eur. J. Org. Chem.*, 2003(6):973–984, March 2003.

[14] Gaspard Huber, Thierry Brotin, Lionel Dubois, Hervé Desvaux, Jean-Pierre Dutasta, and Patrick Berthault. Water Soluble Cryptophanes Showing Unprecedented Affinity for Xenon: Candidates as NMR-Based Biosensors. *J. Am. Chem. Soc.*, 128(18):6239–6246, May 2006.

[15] Matti Hanni, Perttu Lantto, Nino Runeberg, Jukka Jokisaari, and Juha Vaara. Calculation of binary magnetic properties and potential energy curve in xenon dimer: Second virial coefficient of 129xe nuclear shielding. *J. Chem. Phys.*, 121(12):5908–5919, September 2004.

[16] Michal Straka, Perttu Lantto, and Juha Vaara. Toward Calculations of the 129xe Chemical Shift in Xe@C60 at Experimental Conditions: Relativity, Correlation, and Dynamics. *J. Phys. Chem. A*, 112(12):2658–2668, March 2008.

[17] Marcin Selent, Jonas Nyman, Juho Roukala, Marek Ilczyszyn, Raija Oilunkaniemi, Peter J. Bygrave, Risto Laitinen, Jukka Jokisaari, Graeme M. Day, and Perttu Lantto. Clathrate Structure Determination by Combining Crystal Structure Prediction with Computational and Experimental 129Xe NMR Spectroscopy. *Chemistry – A European Journal*, 23(22):5258–5269, 2017.

[18] Sanna Komulainen, Juho Roukala, Vladimir V. Zhivonitko, Muhammad Asadullah Javed, Linjiang Chen, Daniel Holden, Tom Hasell, Andrew Cooper, Perttu Lantto, and Ville-Veikko Telkki. Inside information on xenon adsorption in porous organic cages by NMR. *Chemical Science*, 8(8):5721–5727, July 2017.

[19] ADF 2018, SCM, Theoretical Chemistry, Vrije Universiteit, Amsterdam, The Netherlands, `http://www.scm.com`.

[20] G. te Velde, F. M. Bickelhaupt, E. J. Baerends, C. Fonseca Guerra, S. J. A. van Gisbergen, J. G. Snijders, and T. Ziegler. Chemistry with ADF. *J. Comput. Chem.*, 22:931–967, 2001.

[21] G. Schreckenbach and T. Ziegler. Calculation of nmr shielding tensors using gauge-including atomic orbitals and modern density functional theory. *J. Phys. Chem.*, 99:606, 1995.

[22] S. K. Wolff, T. Ziegler, E. van Lenthe, and E. J. Baerends. Density functional calculations of nuclear magnetic shieldings using the zeroth-order regular approximation (zora) for relativistic effects: Zora nuclear magnetic resonance. *J. Chem. Phys.*, 110:7689, 1999.

[23] Axel D. Becke. A new mixing of Hartree-Fock and local density-functional theories. *J. Chem. Phys.*, 98(2):1372–1377, January 1993.

[24] Chengteh Lee, Weitao Yang, and Robert G. Parr. Development of the Colle-Salvetti correlation-energy formula into a functional of the electron density. *Phys. Rev. B*, 37(2):785–789, January 1988.

[25] E. van Lenthe and E. J. Baerends. Optimized slater-type basis sets for the elements 1-118. *J. Comput. Chem.*, 24:1142, 2003.

[26] J. Autschbach. Magnitude of finite-nucleus-size effects in relativistic density functional computations of indirect nmr nuclear spin–spin coupling constants. *ChemPhysChem*, 10:2274–2283, 2009.

[27] Caver analyst 2.0, is a software tool for analysis and visualization of tunnels and channels in protein structures; available from http://www.caver.cz/.

[28] Kristian Kříž, Martin Nováček, and Jan Řezáč. Non-covalent interactions atlas benchmark data sets 3: Repulsive contacts. *J. Chem. Theory Comput.*, 17(3):1548–1561, 2021. PMID: 33620192.

[29] TURBOMOLE V7.5.1 2021, a development of University of Karlsruhe and Forschungszentrum Karlsruhe GmbH, 1989-2007, TURBOMOLE GmbH, since 2007; available from
`https://www.turbomole.org`.

[30] Yannick J. Franzke and Florian Weigend. Nmr shielding tensors and chemical shifts in scalar-relativistic local exact two-component theory. *Journal of Chemical Theory and Computation*, 15(2):1028–1043, 2019. PMID: 30620588.

[31] Eike Caldeweyher, Sebastian Ehlert, Andreas Hansen, Hagen Neugebauer, Sebastian Spicher, Christoph Bannwarth, and Stefan Grimme. A generally applicable atomic-charge dependent london dispersion correction. *The Journal of Chemical Physics*, 150(15):154122, 2019.

[32] Yannick J. Franzke, Robert Treß, Tobias M. Pazdera, and Florian Weigend. Error-consistent segmented contracted all-electron relativistic basis sets of double- and triple-zeta quality for nmr shielding constants. *Phys. Chem. Chem. Phys.*, 21:16658–16664, 2019.

[33] Jan Gerit Brandenburg, Christoph Bannwarth, Andreas Hansen, and Stefan Grimme. B97-3c: A revised low-cost variant of the b97-d density functional method. *The Journal of Chemical Physics*, 148(6):064104, 2018.

[34] A. Klamt and G. Schüürmann. COSMO: a new approach to dielectric screening in solvents with explicit expressions for the screening energy and its gradient. *J. Chem. Soc., Perkin Trans. 2*, 0(5):799–805, January 1993.

[35] Wenjing Meng, Jack K. Clegg, John D. Thoburn, and Jonathan R. Nitschke. Controlling the Transmission of Stereochemical Information through Space in Terphenyl-Edged Fe4l6 Cages. *J. Am. Chem. Soc.*, 133(34):13652–13660, 2011.
