# Supplementary material for: Hyper-CEST NMR of metal organic polyhedral cages reveals hidden diastereomers with diverse guest exchange kinetics
Source: Nat Commun. 2022 Mar 31;13:1708. doi: 10.1038/s41467-022-29249-w (PMC8971460; doi:10.1038/s41467-022-29249-w)
Supplement: Supplementary file 2 — Description of Additional Supplementary Files [file 41467_2022_29249_MOESM2_ESM.pdf]

Description of Additional Supplementary Files

Supplementary Movie 1: Animation of the changes in z-spectra for cycling through different sample temperatures.

Supplementary Movie 2: Animation showing the selective inversion of one of the three peaks while monitoring the overall signal changes over 350 ms.
